# Indexes of Large Genome Collections on a PC

**DOI:** 10.1371/journal.pone.0109384

**Published:** 2014-10-07

**Authors:** Agnieszka Danek, Sebastian Deorowicz, Szymon Grabowski

**Affiliations:** 1 Institute of Informatics, Silesian University of Technology, Gliwice, Poland; 2 Institute of Applied Computer Science, Lodz University of Technology, Łódź, Poland; University of Queensland, Australia

## Abstract

The availability of thousands of individual genomes of one species should boost rapid progress in personalized medicine or understanding of the interaction between genotype and phenotype, to name a few applications. A key operation useful in such analyses is aligning sequencing reads against a collection of genomes, which is costly with the use of existing algorithms due to their large memory requirements. We present MuGI, Multiple Genome Index, which reports all occurrences of a given pattern, in exact and approximate matching model, against a collection of thousand(s) genomes. Its unique feature is the small index size, which is customisable. It fits in a standard computer with 16–32 GB, or even 8 GB, of RAM, for the 1000GP collection of 1092 diploid human genomes. The solution is also fast. For example, the exact matching queries (of average length 150 bp) are handled in average time of 39* µs* and with up to 3 mismatches in 373* µs* on the test PC with the index size of 13.4 GB. For a smaller index, occupying 7.4 GB in memory, the respective times grow to 76* µs* and 917* µs*. Software is available at http://sun.aei.polsl.pl/mugi under a free license. Data S1 is available at PLOS One online.

## Introduction

About a decade ago, thanks to breakthrough ideas in succinct indexing data structures, it was made clear that a full mammalian-sized genome can be stored and used in indexed form in main memory of a commodity workstation (equipped with, e.g., 4 GB of RAM). Probably the earliest such attempt, by Sadakane and Shibuya [1], resulted in approximately 2 GB sized compressed suffix array built for the April 2001 draft assembly by Human Genome Project at UCSC. (Obtaining low construction space, however, was more challenging, although later more memory frugal, or disk-based, algorithms for building compressed indexes appeared, see, e.g., [2] and references therein.). Yet around 2008, only a few sequenced human genomes were available, so the possibility to look for exact or approximate occurrences of a given DNA string in a (single) genome was clearly useful. Nowadays, when repositories with a thousand or more genomes are easily available, the life scientists' goals are also more ambitious, and it is desirable to search for patterns in large genomic collections. One application of such a solution could be simultaneous alignment of sequencing reads against multiple genomes [3]. Other applications are discussed in the last section.

Interestingly, this is a largely unexplored area yet. On one hand, toward the end of the previous decade it was noticed that the “standard” compressed indexes (surveyed in [4]), e.g. from the FM or CSA family, are rather inappropriate to handle large collections of genomes of the same species, because they cannot exploit well the specific repetitiveness. On a related note, standard compression methods were inefficient for a simpler problem of merely compressing multiple genomes. Since around 2009 we can observe a surge of interest in practical, multi-sequence oriented DNA compressors [5]–[15], often coupled with random access capabilities and sometimes also offering indexed search. The first algorithms from 2009 were soon followed by more mature proposals, which will be presented below, focusing on their indexing capabilities. More information on genome data compressors and indexes can be found in the recent surveys [16]–[18].

Mäkinen *et al*. [19] added index functionalities to compressed DNA sequences: *display* (which can also be called the random access functionality) returning the substring specified by its start and end position, *count* telling the number of times the given pattern occurs in the text, and *locate* listing the positions of the pattern in the text. Although those operations are not new in full-text indexes (possibly also compressed), the authors noticed that the existing general solutions, paying no attention to long repeats in the input, are not very effective here and they proposed novel *self-indexes* for the considered problem.

Claude *et al*. [7] pointed out that the full-text indexes from [19], albeit fast in counting, are rather slow in extracting the match locations, a feature shared by all compressed indexes based on the Burrows–Wheeler transform (BWT) [4]. They proposed two schemes, one basically an inverted index on *q*-grams, the other being a grammar-based self-index. The inverted index offers interesting space-time tradeoffs (on real data, not in the worst case), but can basically work with substrings of fixed length 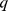. The grammar-based index is more elegant and can work with any substring length, but uses significantly more space, is slower and needs a large amount of RAM in the index build phase. None of these solutions can scale to large collections of mammalian-sized genomes, since even for 37 sequences of S. cerevisiae totaling 428 Mbases the index construction space is at least a few gigabytes.

While a few more indexes for repetitive data were proposed in recent years (e.g., [20]–[23]), theoretically superior to the ones presented above and often handling approximate matches, none of them can be considered a breakthrough, at least for bioinformatics, since none of them was demonstrated to run on multi-gigabyte genomic data.

A more ambitious goal, of indexing 1092 human genomes, was set by Wandelt *et al*. [24]. They obtained a data structure of size 115.7 GB, spending 54 hours on a powerful laptop. The index (loaded to RAM for a single chromosome at a time), called RCSI, allows to answer exact matching queries in about 250* µs*, and in up to 2 orders of magnitude longer time for 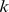-approximate matching queries, depending on the choice of 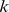 (up to 5).

Sirén *et al*. [25] extended the BWT transform of strings to acyclic directed labeled graphs, to support path queries as an extension to substring searching. This allows, e.g., for read alignment on an extended BWT index of a graph representing a *pan-genome*, i.e., reference genome and known variants of it. The authors built an index over a reference genome and a subset of variants from the dbSNP database, of size less than 4 GB and allowing to match reads in less than 1 ms in the exact matching mode. The structure, called GCSA, was built in chromosome-by-chromosome manner, but unfortunately, they were unable to finish the construction for a few “hard” chromosomes even in 1 TB of RAM! We also note that a pan-genome contains less information than a collection of genomes, since the knowledge about variant occurrences in individual genomes is lost.

A somewhat related work, by Huang *et al*. [26], presents an alignment tool, BWBBLE, working with a multi-genome (which is basically synonymous with pan-genome in the terminology of [25]). BWBBLE follows a more heuristic approach than GCSA and can be constructed using much more humble resources. Its memory use, however, is over 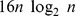 bits, where 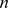 is the multi-genome length. This translates to more than 200 GB of memory needed to build a multi-genome for a collection of 1092 human genomes. Both BWBBLE and GCSA need at least 10 ms to find matches with up to 3 errors.

The recently proposed journaled string tree (JST) [27] takes a different approach, providing an online scan over the reference sequence, but also keeping track of coverages of variants falling into the current window over the reference. Each individual is represented as a journal string, that is, a referentially compressed version of the original sequence; segments of journal strings, together with helper data, are stored in a journal string tree. The JST approach allows to generically speed up many sequential pattern matching algorithms (for exact or approximate search) when working on a collection of similar sequences. A drawback of this approach is that search times are never better than of an online scan over a single (reference) sequence.

Also recently, Durbin [28] presented an interesting data structure dubbed Positional Burrows–Wheeler Transform (PBWT), to find long matches between sequences within a given collection, or between a new test sequence and sequences from the collection. PBWT provides very compact representation of the dataset being searched, yet its application is different to ours: only binary information about variant occurrences are kept (not even their position in a reference sequence), which means that handling standard locate queries (given a string, report all its match positions in the relevant sequences in the indexed collection) is impossible in this way.

Aligning sequencing reads to a genome with possible variants was also recently considered in theoretical works, under the problem name of indexing text with wildcard positions [29], [30], where the wildcards represent SNPs. No experimental validation of the results was presented in the cited papers.

Most of the listed approaches are traditional string data structures, in the sense that they can work with arbitrary input sequences. The nowadays practice, however, is to represent multi-genome collections in repositories as basically a single reference genome, plus a database of possible variants (e.g., SNPs), plus information on which of the variants from the database actually occur in each of the individual genomes. The popular VCF (Variant Call Format) format allows to keep more information about a sequenced genome than listed here, but this minimal collection representation is enough to export each genome to its FASTA form. Dealing with input stored in such compact form should allow to build efficient indexes much more easily than following the standard “universal” way, not to say about tremendous resource savings in the index construction.

This modern approach was initiated in compression-only oriented works [5], [13], [14], and now we propose to adapt it in construction of a succinct and efficient index. According to our knowledge, this is the first full-text index capable to work on a scale of thousand(s) of human genomes on a PC, that is, a small workstation equipped with 16–32 GB of RAM. What is more, for a price of some slow-down the index can be used even on an 8 GB machine. No matter the end of the space-time tradeoff we are, the index is capable of handling also approximate matching queries, that is, reporting patterns locations in particular genomes from the collection with tolerance for up to 5 mismatches. As said, the index is not only compact, but also fast. For example, if up to 3 errors are allowed, the queries are handled in average time of 373* µs* on the test PC and the index takes 13.4 GB of memory, or in 917* µs* when the index is of size 7.4 GB. The current version of our index requires more resources (from 38 GB to 47 GB of RAM, depending on the index settings) in the construction phase; a drawback which may be eliminated in a future work, as discussed in the last section of this paper.

## Materials and Methods

### Datasets

We are indexing large collection of genomes of the same species, which are represented as the reference genome in FASTA format together with the VCF [31] file, describing all possible reference sequence variations and the genotype information for each of the genome in the dataset. We are only interested in details allowing for the recovery of the DNA sequences, all non-essential fields are ignored. Therefore, the data included in the VCFmin format, used in [14], are sufficient. Each line describes a possible variant that may be a single nucleotide polymorphism (SNP), a deletion (DEL), an insertion (INS) or a structural variation (SV), which is typically a combination of a very long deletion and an insertion. The genotype of each genome is specified in one designated column with information if each of the variant is found in this genome. In case of diploid and phased genotypes this information concerns two basic, haploid chromosome sets for each genome and treats them independently. Thus for any phased diploid genome, its DNA sequence is twice the size the reference sequence.

In our experiments we used the data available from Phase 1 of the 1000 Genomes Project [32] describing the collection of 1092 phased human genomes. We concatenated the available 24 VCF files (one for each chromosome), to get one combined VCF file, which—together with the reference sequence—is the input of our algorithm building the index.

### The general idea

Our tool, Multiple Genome Index (MuGI), performs fast approximate search for input patterns in an indexed collection of genomes of the same species. The searched patterns can be provided in a text file (one pattern per line), or in FASTA or FASTQ format. The index is built based on the reference genome and the VCF file describing the set. The search answers the locate query—the result consists of all positions of the pattern with respect to the reference genome along with the list of all individuals in which it can be found.

The basic search regime is exact matching. Its enhanced version allows for searching with mismatches. Both modes use the seed-and-extend scheme. The general mechanism is to quickly find a substring of the pattern and then extend this seed to verify if it answers the query.

The index has one construction-time parameter, 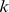, which is the maximum possible length of the seed. The match can be found directly in the reference genome and/or in its modified form, with some of the variations introduced. To find the seed we build an array of all possible 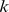-length sequences (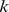-mers) occurring in all genome sequences. In the space-efficient version only a part of the array is kept. The extension step is done using the reference and the available database of variants, checking which combination of possible variations introduced, if any, allows to find the full pattern.

To know individuals in which the match can be found, we have to identify all variants whose occurrence, or absence of, have impact on the match, and list only the genomes with such combination of variants.

### Building the index

To build the index, we process the input data to create the following main substructures, described in detail in the successive paragraphs:

- the reference sequence (REF),
- the Variant Database (VD),
- the Bit Vectors (BVs) with information about variants in all genomes,
- the 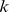-Mer Array (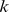
MA) for all unique 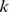-length sequences in the set.

REF is stored in compact form, where 4 bits are used to (conveniently) encode a single character.

VD contains details about all possible variations. For each variant, the following items are stored: type (1 byte), preceding position (4 bytes) and alternative information (4 bytes). (Note that we keep the preceding positions to be able to manage the variants INSs, DELs and SVs, as this convention conforms to their description in VCF files.) The last item indicates alternative character in case of SNP, length of the deletion in case of DEL and position in the additional arrays of bytes (VD-aux) in case of INS and SV. VD-aux holds insertion length (4 bytes) and all inserted characters (1 byte each), if any, for every INS and SV. For SV it also stores length of the deletion (4 bytes). The variants are ordered by the preceding position and a lookup table is created to accelerate search for a variant by its location. VD together with REF can be used to decode the modified sequence from some given position to the right, by introducing certain variants. To be able to decode the sequence to the left, an additional list of all deletions (SVs and DELs), ordered by the resulting position, is created. The list, VD-invDel, stores for each variant its number in the main VD (4 bytes) and the resulting position, that is, the position in the reference after the deletion (4 bytes).

There is one BV for each variant, each of size of the number of genomes in the collection (2 times the number of genomes for diploid organisms). Value 1 at some 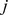 th position in this vector means that the current variant is found in the 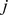 th haploid genome. To reduce the required size, while preserving random access, we keep the collection of these vectors in compressed form, making use of the fact that spatially close variant configurations are often shared across different individuals. The compression algorithm makes use of a dictionary of all possible unique 192-bit chunks (the size chosen experimentally). Each BV is thus represented as a concatenation of 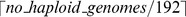 4-byte tokens (vocabulary IDs).


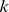
MA keeps information about each 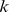-length sequence (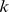-mer) occurring in the whole collection of genomes. The 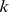-mer sequence itself is not kept. Instead, only the minimum information needed to retrieve it with help of REF and VD is stored. Based on the amount of details necessary to keep, we partition 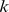-mers into four groups, each stored in one of the four subarrays of kMA: 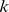
MA^0^, 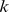
MA^1^, 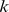
MA^2^ or 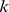
MA^3^. The entries in each subarray are sorted according to the lexicographical order of 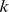-mers they represent. All 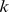-mers beginning with the unknown character (i.e., N or n) are filtered out.

All 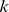-mers found in REF are kept in 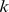
MA^0^. Only the preceding position 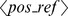 (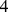 bytes) is stored for each such 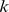-mer, as it is enough (using REF) to retrieve its sequence. These 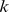-mers are present in all genomes with no variants introduced in the corresponding segment.

The 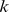-mers that are obtained by applying some variant to the reference sequence are stored in 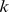
MA^1^/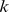
MA^2^/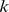
MA^3^. They are produced with going through the reference genome and checking for each position 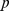 if there is any possible variant with the preceding position in the range from 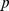 to 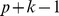. If the check is positive, we decode the 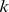-mer. The decoding process takes into account all possible *paths*. By *path* we understand any combination of occurrence of subsequent variants, influencing the decoded sequence. For example, if SNP is possible at current position (i.e., it is listed in VD), two paths are considered: when it is found and when it is absent, resulting in two decoded sequences, differing in the last inspected character. Thus, starting from a single preceding position, many resulting sequences may be obtained. To decode most 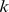-mers, it is enough to store the preceding position plus flags about the presence/absence of following variants. This evidence list (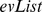) is stored as a bit vector, where 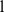 means that the corresponding variant is present. For any 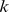-mer starting inside an insertion (INS or SV) it is also necessary to store the 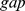 from the beginning of the inserted string to the first character of the 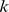-mer.

The 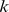-mer with no 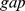 and at most 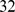 evidences about consecutive variants from VD in the 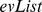 is stored in 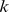
MA^1^, where each entry is defined as 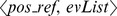 (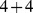 bytes). If there is also a 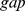 involved, such 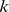-mer goes to 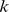
MA^2^, defining each entry as 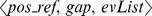 (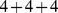 bytes). All 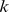-mers with more than 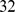 evidences in the 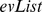 or with evidences about nonconsecutive (with respect to VD) variants are kept in 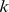
MA^3^, where each 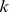-mer is represented by four fields: 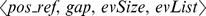 (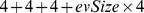 bytes). The representative example of the latter case is a 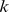-mer with SV introduced and many variants in VD placed within the deleted region. Keeping track of these variants, not altering the resulting sequence, is pointless.

Any 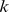-mer is kept in 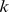
MA only if there is at least one haploid genome that includes it, that is, has the same combination of occurring variants. It is checked with help of BV. Recall that the 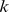-mers in each subarray 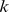
MA*^i^*, 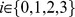, are sorted lexicographically. To speed up the binary search (by narrowing down the initial search interval), a lookup table, taking into account the first 12 characters, is created for each subarray.

### The basic search algorithm

The pseudocode of the basic search algorithm is presented as Algorithm 1 in Table 1. It looks for all exact occurrences of the pattern 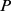 in the compressed collection, using the seed-and-extend scheme. The undetermined nucleotides (i.e., N or n) occurring in 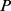 are encoded differently than in REF, so they never match any character in the collection. The seed 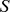 is chosen to be a substring of 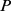, precisely its first 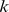 characters, or the full pattern, if 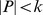 (lines 1–2).

**Table 1:** Pseudocode of the basic search algorithm.

Algorithm 1 exactSearch(*P*)
{ 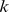 *MA, vtList and evList are global variables*}
1 *p* ← min(|*P*|, 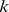 )
2 *S* ← substring(*P*, 0, *p* −1) {*Retrieving the seed S*}
3 **for** *i* ← 0 **to** 3 **do**
4 (*ℓ*, *r*) ← binSearch( 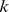 MA*^i^*, *S*) {*Locating the seed S*}
5 **for** *j* ← *ℓ* **to** *r* **do**
6 (*vtList*, *evList*, *pos_curr*, *vt_curr*) ← partDecode( 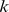 MA*^i^*[*j*], *p*)
7 extend(*P*, *p*, 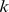 MA*^i^*[*j*].*pos_ref*, *pos_curr*, *vt_curr*) {*Extending the seed S to find P locations*}

The first step is to scan 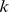
MA for all 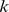-mers whose prefixes (or simply full sequences, if 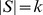) match 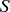. It is done with binary search in each subarray 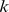
MA*^i^*, 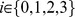, separately (lines 3–4). Next, each found seed is partly decoded and then extended (lines 5–7). The partial decoding, done by the 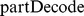 function, starts from 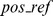 of the current 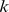-mer and move 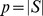 characters forward, according to the 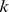-mer's details (i.e., there may be a need to introduce some found variant). Character-by-character matching is not performed, as it is already known that 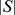-length prefix of the 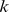-mer matches 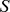. Function 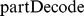 returns the seed's succeeding position (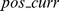) and variant (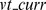) in the reference, along with the list of encountered variants (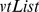) and the list of evidences about their presence or absence (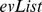). The latter is a vector of 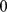 s in case of 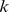
MA^0^ and a copy of 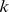-mer's 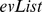 (or its part) for other subarrays. The first variant (the one with preceding position greater than or equal to the preceding position of the 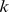-mer) is found with binary search in VD. It is not shown in the pseudocode, but for each seed also the preceding SVs and DELs are taken into account when creating the initial 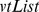 and 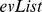.

The seed 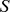 is recursively extended according to all possible combinations of variants, that is, as long as succeeding characters match the characters in 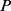 and found occurrences of 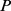 are reported (line 7). The pseudocode of the algorithm extending the seed and reporting the results is presented as Algorithm 2 in Table 2. Maintained variables are: full pattern 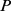, 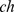 (number of decoded characters), 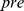 and 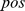 (the preceding position of the seed and the current position, both in relation to the reference), and 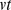 (next variant from VD). Also 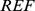, 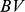, the current 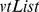 and 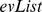 are available. If position of 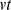 (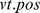) is greater than 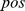 (lines 2–5), no variant is introduced and the next character is taken from REF. If it does not match the related character in 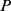, the extension is stopped, as the current path is not valid. If 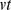 is encountered at 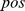 (lines 6–11), it is added to the 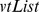 and two paths are checked—when it is introduced (new bit in 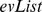 is set to 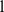) and when it is not (new bit in 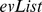 is set to 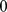). The first path is not taken if 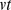 does not match 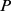. It can happen for SNPs and inserted characters (from INS or SV). If 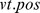 is less than 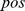 (lines 12–19), it means 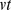 is placed in region previously deleted by other variant. The only possibility that 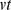 is taken into account is if it deletes characters beyond previous deletion. Otherwise it is skipped.

**Table 2:** Pseudocode of the algorithm extending the found seed in the basic search.

Algorithm 2 extend(*P*, *ch*, *pre*, *pos*, *vt*)
{*REF*, *BV*, *vtList and evList are global variables*}
1 **while** *ch* < |*P*| **do**
2 **if** *vt.pos* > *pos* **then** {*No variant at pos*}
3 **if** *REF*[*pos*] = *P*[*ch*] **then**
4 *pos* ← *pos* +1; *ch* ← *ch* +1
5 **else** report **false **{*Invalid path*}
6 **else if** *vt.pos* = *pos* **then**
7 *vtList*.add(*vt*); *evList*.add(*1*);
8 **if** *vt* matches *P* **then**
9 *new* ← *pos* + *vt.delLen*
10 extend(*pre*, *new*, *ch* + *vt.len*, *vt* +1)
11 *evList*.setLast(*0*); *vt* ← *vt* +1
12 **else **{*vt.pos* < *pos*}
13 *new* ← *vt.pos* + *vt.delLen*
14 **if** *new* > *pos* **then**
15 *vtList*.add(*vt*); *evList*.add(*1*);
16 **if** *vt* matches *P* **then**
17 extend(*pre*, *new*, *ch* + *vt.len*, *vt* +1)
18 *evList*.setLast(*0*)
19 *vt* ← *vt* +1
20 *R* ← 1*^noHaploidGenomes^* {*a bit-vector of noHaploidGenomes bits 1*}
21 **for** *i* ← 1 **to** *vtList.size* **do**
22 **if** *evList*[*i*] **then** *R* ← *R* & *BV*[*i*]
23 **else** *R* ← *R* & ∼*BV*[*i*]
24 **if** *R* = 0 **then** report **false** {*Invalid path*}
25 **else** report (*pre*, *R*) {*P found*}

When the extension reaches the end of the pattern 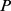, it is checked in which individuals, if in any, the relevant combination of variants (track kept in 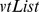) is found (lines 20–25). The bit vector 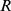 is initialized to be the size of the number of haploid genomes. The value 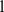 at 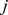 th position means that 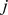 th haploid genome contains the found sequence. The vector 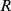 is set to all 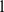 s at the beginning, because if 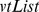 is empty, the sequence is present in all genomes. To check which genomes have the appropriate combination of variants, the bitwise AND operations are performed between all BVs related to variants from the 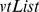, negating all BVs with 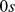 at the corresponding position in the 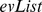. If 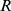 contains any 1 s, pattern 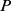 is reported to be found with the preceding position 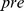 (in relation to the reference genome) and vector 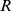 specifies genomes containing such sequence.

### The space-efficient version

To reduce the required space, while still being able to find all occurrences of the pattern, we make use of the idea of sparse suffix array [33]. This data structure stores only the suffixes with preceding position being a multiple of 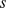 (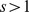 is a construction-time parameter). In our scheme, the two largest subarrays, 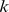
MA^0^ and 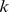
MA^1^, are kept in sparse form, based on preceding positions of 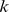-mers. For 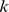
MA^1^, it is also necessary to keep all 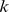-mers that begin with deletion or insertion (the first variant has the same preceding position as the 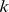-mer).

The search algorithm has to be slightly modified. Apart from looking for the 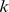-length prefix of the pattern (i.e., 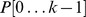) in 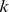
MA, also 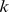-length substrings starting at positions 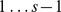 must be looked for in 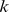
MA^0^, 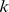
MA^1^, and 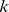
MA^3^ (as some specific seeds may be present only in 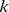
MA^3^). The substrings, if found in one of mentioned subarrays, must be then decoded to the left, to check if their prefix (from 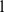 to 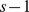 characters, depending on the starting position) matches the pattern 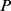. The VD-invDel substructure is used for the process. The rest of search is the same as in the basic search algorithm.

### The approximate search algorithm

The approximate search algorithm looks for all occurrences of the given pattern with some maximum allowed number of mismatches. According to the well-known property, for any sequence of length 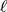 with 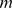 mismatches at least one of the consecutive substrings of length 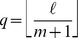 is the same as in the original sequence. Therefore, the approximate search begins with dividing the string to 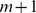 substrings of length 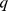. Next, the exact search algorithm is used to look for each of the substrings. If a substring is found in the collection, it is further decoded to the right and to the left, similarly as in the exact search, but allowing for at most 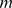 differences between the decoded sequence and the searched sequence. Expanding to the left is done with aid of the same auxiliary substructure as in the space-efficient version (VD-invDel). The list of genomes in which the found sequences are present is obtained in the same way as in the exact searching.

### Test data

To evaluate the algorithm, we first used a similar methodology as the one in [24]. To this end, we generated a file with 100K queries, where each pattern is a modified excerpt of length 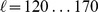 (uniformly random value) from a randomly selected genome from the collection, starting at a randomly selected position. Excerpts containing undetermined nucleotide (i.e., N) were rejected. The modifications consisted in introducing random nucleotides in place of 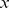 existing nucleotides, where 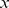 is a randomly selected integer number from the 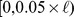 range.

Additionally, we use real reads from the 1000GP repository. There are 140K reads chosen randomly in such a way that each of 14 human populations is represented with 10K reads. Their length varies between 100 and 120 bp. Both data sets are available at project home page.

As the index construction costs are not that small (as mentioned earlier), we provide an exemplary index over the 1000GP data at our software page.

## Results

All experiments were performed on a PC with Intel Core i7 4770 3.4 GHz CPU (4 cores with hyperthreading), equipped with 32 GB of RAM, running Windows 7 OS. The C++ sources were compiled using GCC 4.7.1 compiler.

The index was built on another machine (2.4 GHz Quad-Core AMD Opteron CPU with 128 GB RAM running Red Hat 4.1.2-46) and required more RAM: from 38 GB (for 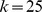) to 47 GB (for 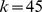). The corresponding build times were 15 hours and 72 hours, respectively. The index build phase was based on parallel sort (using Intel TBB and OpenMP libraries), while all the queries in our experiments were single-threaded. The correctness of obtained query results, in exact and approximate matching mode, was experimentally verified with a set of patterns, for which a naïve (sequential) scan over all the sequences was run.

**Figure 1:**
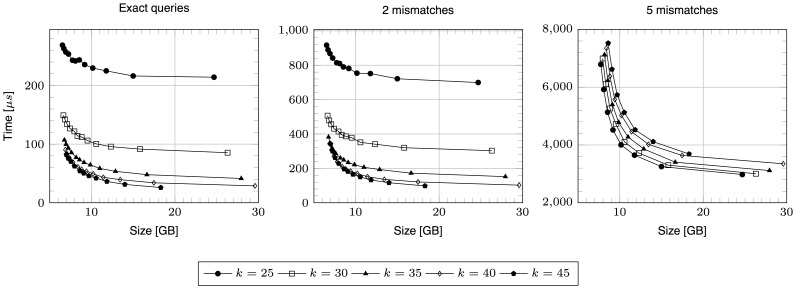
Average query times vs. index sizes. Simulated reads were used.

From Table 3 we can see that the fastest index version (i.e., with sparsity 1, which translates to standard 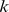-mer arrays) may work on the test machine even for the seed maximum length of 40 symbols. Significant savings in the index size are however possible if sparsity of 3 or more is set, making the index possible to operate on a commodity PC with 16 GB of RAM. If one (e.g., a laptop user) requires even less memory, then the sparsity set to 16 makes it possible to run the index even in 8 GB of RAM. Naturally, using larger sparsities comes at a price of slower searches; in Fig. 1, each series of results for a given value of 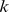 corresponds to sparsities from 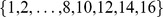 (sparsities of 1 correspond to the rightmost points, with the exception of the case of 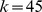, for which the sparsities start from 2). Still, this tradeoff is not very painful: even the largest allowed sparsity value (16) slows down the fastest (for sparsity of 1) queries by factor about 2 on average, in most cases.

**Table 3:** Index sizes.

Sparsity	Size [GB]				
	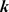 = 25	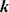 = 30	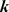 = 35	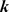 = 40	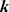 = 45
1	24.7	26.3	27.9	29.6	31.2
2	15.0	15.8	16.6	17.5	18.3
3	11.8	12.3	12.9	13.4	14.0
4	10.2	10.6	11.0	11.4	11.8
5	9.2	9.5	9.9	10.2	10.5
6	8.5	8.8	9.1	9.4	9.7
7	8.1	8.3	8.6	8.8	9.1
8	7.7	7.9	8.2	8.4	8.6
10	7.2	7.4	7.6	7.8	8.0
12	6.9	7.1	7.2	7.4	7.5
14	6.7	6.8	7.0	7.1	7.2
16	6.5	6.6	6.8	6.9	7.0

Costlier, in terms of query times, is handling mismatches. In particular, allowing 4 or 5 mismatches in the pattern requires at least an order of magnitude longer query times than in the exact matching mode. Yet, even for 5 allowed errors the average query time was below 10 ms in all tests. This translates, for example, to 224 mapped reads per second allowing up to 5 mismatches and 10,593 mapped reads per second with up to 1 mismatch, at index size of 11.4 GB (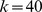, sparsity of 4, simulated reads; cf. Table 4).

**Table 4:** Query times for various variants of indexes for simulated data.

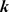	*sparsity*	size	Max. allowed mismatches					
		[GB]	0	1	2	3	4	5
25	1	24.7	214.2	450.8	699.5	971.5	1,438.3	2,976.8
25	3	11.8	225.0	481.2	751.6	1,024.3	1,599.9	3,647.1
25	4	10.2	229.8	493.1	754.0	1,050.9	1,676.4	4,004.2
25	8	7.7	243.1	528.3	814.6	1,158.5	2,341.4	6,790.4
25	12	6.9	257.2	558.3	868.0	1,337.8		
25	16	6.5	268.8	588.6	916.6	1,787.9		
30	1	26.3	85.4	193.4	303.0	456.0	1,036.4	3,004.6
30	3	12.3	95.7	220.8	340.6	520.4	1,258.0	3,716.2
30	4	10.6	100.4	227.8	351.5	544.0	1,376.5	4,104.5
30	8	7.9	121.8	267.0	414.5	713.6	2,215.2	6,994.5
30	12	7.1	134.0	291.4	456.4	959.4		
30	16	6.6	149.2	319.3	506.8	1,490.4		
35	1	27.9	41.4	98.0	152.0	301.8	1,033.4	3,114.6
35	3	12.9	53.6	121.2	193.0	380.4	1,280.8	3,861.2
35	4	11.0	58.6	130.2	206.3	419.2	1,411.7	4,277.6
35	8	8.2	77.2	166.1	260.3	608.3	2,224.4	7,120.4
35	12	7.2	93.3	196.2	314.6	905.3		
35	16	6.8	107.0	222.2	382.4	1,506.4		
40	1	29.6	28.8	65.2	102.3	291.0	1,109.9	3,348.8
40	3	13.4	39.4	85.5	136.1	372.5	1,334.0	4,021.0
40	4	11.4	43.4	94.4	151.4	412.2	1,471.1	4,461.1
40	8	8.4	61.0	128.9	210.3	615.4	2,297.9	7,350.3
40	12	7.4	76.3	160.0	271.8	917.0		
40	16	6.9	90.4	184.4	344.3	1,514.1		
45	2	18.3	25.9	56.2	97.9	329.6	1,207.0	3,687.2
45	3	14.0	31.3	67.7	116.5	375.5	1,353.3	4,115.0
45	4	11.8	36.3	77.2	132.6	421.2	1,490.9	4,525.3
45	8	8.6	54.2	112.4	196.2	625.8	2,394.4	7,523.9
45	12	7.5	70.4	142.9	262.5	942.0		
45	16	7.0	82.1	168.4	342.3	1,531.9		
GEM mapper		5.0	24.0	50.6	64.9	86.4	131.0	217.3

All times in *µs*. We do not provide times for large sparsities and more errors than 3, since in such cases the internal queries would be for very short sequences and in turn result in numerous matches and significant times; thus, we do not recommend to use MuGI in such parameter configurations.

**Figure 2:**
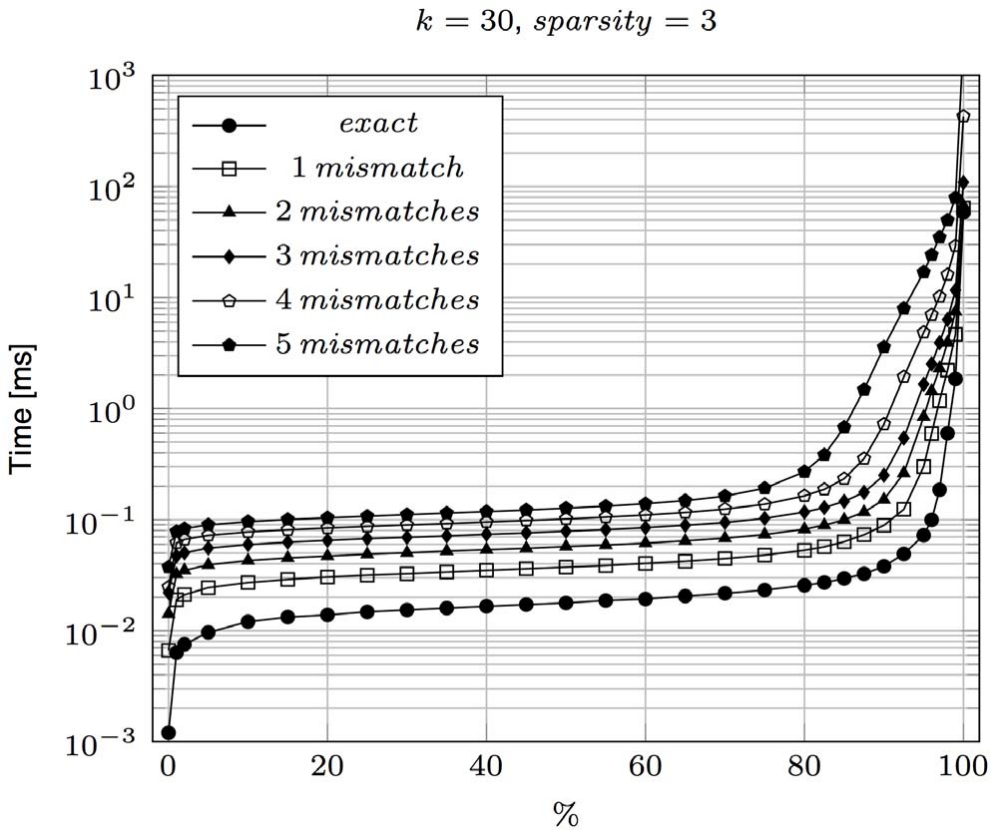
Query time percentiles for exact and approximate matching, for max error up to 5. For example, the 80th percentile for 1 error equal to 0.52 ms means that 80% of the test patterns were handled in time up to 0.52 ms each, allowing for 1 mismatch. Simulated reads were used.

Apart from the average case, one is often interested also in the pessimistic scenario. Our search algorithms do not have interesting worst-case time complexities, but fortunately pathological cases are rather rare. To measure this, for each test scenario a histogram of query times over 100K patterns was gathered, and the time percentiles are shown in Fig. 2. Note that the easy cases dominate: for all maximum errors allowed, for 90% test patterns the query time is below the average. Yet, there are a few percent of test patterns for which the times are several times longer, and even a fraction of a percent of patterns with query times exceeding 100 ms (at least for approximate matching). More details exposing the same phenomenon are presented in Table 5.

**Table 5:** Query times for simulated data for 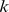 = 40 and *sparsity*  = 3 (size 13.4 GB).

Percentile	Max. allowed mismatches					
	0	1	2	3	4	5
10%	12.4	27.7	44.0	62.1	81.1	100.0
25%	15.4	32.2	50.3	69.9	90.7	111.8
50%	18.7	38.3	58.8	81.4	105.8	131.4
75%	23.8	48.5	73.5	103.7	141.9	199.5
90%	35.9	76.5	116.0	188.0	707.0	3,747.5
95%	57.0	112.7	182.9	718.1	4,972.8	17,619.4
average	39.4	85.5	136.1	372.5	1,334.0	4,021.0

All times in *µs*.

While we cannot directly compare our solution to RCSI by Wandelt *et al*. [24], as their software is not public, we can show some comparison. Their index was built over twice less data (haploid human genomes vs. diploid genomes in our data). We handle exact matches much faster (over 6 times shorter reported average times, but considering the difference in test computers this probably translates to factor about 4). Roughly similar differences can be observed for the approximate matching scenario, but RCSI handles the Levenshtein distance, while our scheme handles (so far) only mismatches. Finally, and perhaps most importantly, our index over 1092 diploid human genomes can be run on a standard PC, equipped with 32 or 16 GB of RAM (or even 8 GB, for the price of more slow-down), while RCSI requires a machine with 128 GB (unless searches are limited to one chromosome, when a portion of the index may be loaded into memory).

We were are not able to run GCSA [25] or BWBBLE [26], due to their large memory requirements in the construction phase.

We did, however, ran a preliminary comparison of MuGI against GEM [34], one of the fastest single genome read mappers. We ran it on 1 CPU core, for mismatches only, in the all-strata mode, in which all matches with 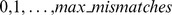 errors are reported, in arbitrary order. Table 4 contains a detailed rundown of the results on simulated reads. For example, we can see that GEM performed exact matching in 24.0 *µs*, found matches with up to 1 mismatch in 50.6 *µs*, matches with up to 3 mismatches in 86.4 *µs*, and matches with up to 5 mismatches in 217.3 *µs*. The memory use was 5.0 GB. This means that, depending on chosen options of our solution, GEM was only about twice faster in the exact matching mode and 15–20 times faster when 5 mismatches were allowed. On real reads (Table 6) GEM is about 1.5–5 times faster with exact matching and about 10–20 times faster with 3 allowed mismatches. The major scenario difference is however that GEM performs mapping to a single (i.e., our reference) genome, so to obtain the same mapping results GEM would have to be run 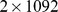 times, once per haploid genome. We thus consider these preliminary comparative results very promising.

**Table 6:** Query times for various variants of indexes for real data.

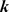	*sparsity*	size	Max. allowed mismatches				
		[GB]	0	1	2	3	4
25	1	24.7	214.1	483.2	741.6	1,123.3	3,566.8
25	3	11.8	227.6	509.4	791.1	1,271.6	4,575.3
25	4	10.2	238.8	520.9	795.0	1,375.8	5,134.4
25	8	7.7	259.1	569.2	865.3	2,180.1	9,634.0
25	12	6.9	282.0	605.6	960.4	4,052.4	
25	16	6.5	292.6	644.0	1,264.0	9,888.8	
30	1	26.3	93.9	206.2	342.4	938.8	3,652.4
30	3	12.3	105.8	234.5	384.8	1,191.8	4,691.9
30	4	10.6	111.2	241.7	386.8	1,310.5	5,310.1
30	8	7.9	131.3	283.9	485.4	2,183.5	9,862.3
30	12	7.1	149.2	316.0	674.2	4,075.0	
30	16	6.6	161.1	343.7	1,051.7	10,128.0	
35	1	27.9	51.5	109.5	200.2	977.1	3,782.0
35	3	12.9	62.6	132.4	255.7	1,267.1	5,224.2
35	4	11.0	75.2	156.7	307.5	1,491.0	5,977.3
35	8	8.2	94.3	193.3	463.7	2,434.8	9,893.8
35	12	7.2	98.9	206.7	630.0	4,099.5	
35	16	6.8	113.2	230.7	1,018.4	10,267.9	
40	1	29.6	34.1	68.1	191.4	1,004.6	3,793.7
40	3	13.4	43.3	90.6	250.3	1,248.2	4,878.4
40	4	11.4	49.3	100.2	280.1	1,375.3	5,491.2
40	8	8.4	67.2	134.4	426.2	2,282.5	10,236.1
40	12	7.4	80.7	165.3	645.2	4,240.8	
40	16	6.9	95.1	193.3	1,055.9	10,497.4	
45	2	18.3	30.0	61.3	219.2	1,116.7	4,327.8
45	3	14.0	35.5	72.7	259.0	1,281.7	4,988.0
45	4	11.8	40.7	82.2	283.5	1,401.1	5,598.8
45	8	8.6	58.3	118.4	432.1	2,320.6	10,420.5
45	12	7.5	73.4	149.9	657.3	4,289.5	
45	16	7.0	86.4	182.4	1,072.4	10,647.0	
GEM mapper		5.0	22.1	56.5	78.5	126.2	221.6

All times in *µs*. We do not provide times for large sparsities and more errors than 3, since in such cases the internal queries would be for very short sequences and in turn result in numerous matches and significant times; thus, we do not recommend to use MuGI in such parameter configurations.

Finally, in Table 7 we compare MuGI against a recent tool JST by Rahn *et al.* [27]. As we can see, MuGI is usually 5–6 orders of magnitude faster at somewhat less memory consumption. This huge gap in performance can be explained with two different search “philosophies”: sequential scan over the reference sequence in JST vs. fully indexed search in MuGI. As in this test we used only chr1 data (1092 sequences), the performance gap would probably be larger with the full human collection. On the other hand, we admit that JST performance with growing 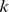 (the maximum allowed number of errors) remains unchanged (which is a property of Myers' algorithm), therefore this scheme might be a satisfactory choice for a collection of short and highly-varied genomes.

**Table 7:** Comparison of MuGI and JST on simulated and real data, both over 1092 individual sequences of chr1.

Algorithm	Max. allowed mismatches						RAM usage
	0	1	2	3	4	5	[GB]
**Simulated data**							
JST-Horspool	8.0 s	—	—	—	—	—	2.58
JST-Myers	22.5 s	24.0 s	23.9 s	24.3 s	24.5 s	24.9 s	2.58
MuGI, 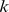 = 30, *sparsity* = 1	8.2* µs*	14.8* µs*	22.7* µs*	33.6* µs*	69.6* µs*	176.0* µs*	1.84
MuGI, 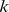 = 30, *sparsity* = 3	10.7* µs*	21.7* µs*	32.4* µs*	47.9* µs*	90.7* µs*	239.0* µs*	0.98
**Real data**							
JST-Horspool	6.9 s	—	—	—	—	—	2.58
JST-Myers	18.4 s	19.1 s	19.2 s	20.0 s	20.3 s	20.3 s	2.58
MuGI, 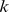 = 30, *sparsity* = 1	7.6* µs*	14.3* µs*	22.1* µs*	53.4* µs*	172.3* µs*	476.5* µs*	1.84
MuGI, 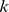 = 30, *sparsity* = 3	12.3* µs*	23.1* µs*	35.3* µs*	72.0* µs*	238.2* µs*	617.9* µs*	0.98

MuGI was executed for parameters 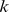 = 30 and sparsities: 1 (index size 2.0 GB), 3 (index size 1.0 GB). Results for JST are averages from only 100 queries due to very long running times. JST times include block generation (blocks of 100K SNPs were used), but in our experiments they are at least an order of magnitude lower than pattern searching. JST-Horspool uses the Boyer–Moore–Horspool exact matching algorithm, while JST-Myers uses Myers' bit-parallel approximate matching algorithm, handling the Levenshtein distance (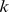-differences). The JST index size was 468 MB, in addition to the 253 MB of the reference sequence. Note its memory use during the search is significantly higher than the index size and depends on the block size (e.g., its memory use grows to about 13 GB with blocks of 1 M SNPs).

## Discussion

We presented an efficient index for exact and approximate searching over large repetitive genomic collections, in particular: multiple genomes of the same species. This has a natural application in aligning sequencing reads against a collection of genomes, with expected benefits for, e.g., personalized medicine and deeper understanding of the interaction between genotype and phenotype. Experiments show that the index built over a collection of 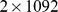 human genomes fits a PC machine with 16 GB of RAM, or even half less, for the price of some slow-down. According to our knowledge, this is the first feat of this kind. The obtained solution is capable of finding all pattern occurrences in the collection in much below 1 ms in most use scenarios.

We point out that representing a “true” genome as a linear sequence over the ACGT(N) alphabet is inherently imperfect, since our knowledge about these sequences is (and will likely remain in the near future) limited. Every sequencing technology introduces its errors, therefore storing qualities (i.e., *estimated* correctness probabilities) together with the DNA symbols would convey more information useful for read mapping, yet we are unable to imagine an index over large collections based on such information not requiring huge amount of resources (especially main memory) in its runtime and construction stages. Moreover, large discrepancies between the reference and a given genome, e.g., long indels, result in reads that cannot be usually mapped, which implies incomplete variant information in the built VCF. Basically for those reasons the application of MuGI (and related software, like RCSI or BWBBLE) for mapping sequencing reads trades some accuracy for performance and reasonable memory use, yet with improving sequencing technologies the obtained mapping results should also be more valuable.

On the other hand, we should stress that MuGI is an index rather than a full-fledged read mapper. Aligning reads to multiple genomes is one of its possible applications. Another example could be searching for nullomers, that is, 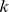-mers with no occurrences in a given genome (or, in our scenario, genome collection). To apply MuGI here, we may generate random strings of specified length (e.g., 20) in a loop and check if they have any occurrence; we may also force the mimimum distance to any 20-mer in the genome to be 2 or 3, with running the MuGI engine in the approximate matching mode, to minimize the impact of noisy data in a genome, at still acceptable search speed. Also a closely related problem of finding the minimal absent word was investigated in the literature, and it can be solved with MuGI with a systematic scan over its component structures. Nullomers/minimal absent words can be used for studies of population genetics, drug discovery and development, evolution studies, design of molecular barcodes or specific adaptors for PCR primers [35], [36]. Other (or more general) areas for application of our algorithm may include comparative genomics and personalized medicine.

Several aspects of the presented index require further development. The current approximate matching model comprises mismatches only; it is desirable to extend it to edit distance. The pathological query times could be improved with extra heuristics (even if it is almost irrelevant for large bulk queries). A more practical speedup idea is to enhance the implementation with multi-threading. Some tradeoffs in component data structures (cf. Table 8) may be explored, e.g., the reference genome may be encoded more compactly but at a cost of somewhat slower access. A soft spot of the current implementation is the index construction phase, which is rather naïve and can be optimized especially towards reduced memory requirements. We believe that existing disk-based suffix array creation algorithms (e.g., [37]) can be adapted for this purpose. Alternatively, we could build our indexing data structure separately for each chromosome (with memory use for the construction reduced by an order of magnitude) and then merge those substructures, onto disk, using little memory. The sparse suffix array may be replaced with a sampled suffix array variant [38], for a hopefully faster search at a similar space consumption. Finally, experiments on other collections should be interesting, particularly on highly-polymorphic ones.

**Table 8:** Sizes of the index components.

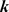	*sparsity*	REF	VD	BV	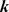 MA^0^	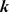 MA^1^	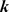 MA^2^	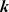 MA^3^	Total
25	1	1,548	698	2,704	11,502	8,021	84	123	24,680
25	3	1,548	698	2,704	3,879	2,731	84	123	11,767
25	4	1,548	698	2,704	2,926	2,070	84	123	10,153
25	8	1,548	698	2,704	1,496	1,078	84	123	7,732
25	12	1,548	698	2,704	1,020	747	84	123	6,925
25	16	1,548	698	2,704	782	582	84	123	6,521
30	1	1,548	698	2,704	11,502	9,634	85	137	26,307
30	3	1,548	698	2,704	3,879	3,270	85	137	12,320
30	4	1,548	698	2,704	2,926	2,474	85	137	10,571
30	8	1,548	698	2,704	1,496	1,281	85	137	7,948
30	12	1,548	698	2,704	1,020	883	85	137	7,074
30	16	1,548	698	2,704	782	684	85	137	6,637
35	1	1,548	698	2,704	11,502	11,254	85	151	27,942
35	3	1,548	698	2,704	3,879	3,810	85	151	12,875
35	4	1,548	698	2,704	2,926	2,880	85	151	10,992
35	8	1,548	698	2,704	1,496	1,484	85	151	8,167
35	12	1,548	698	2,704	1,020	1,019	85	151	7,225
35	16	1,548	698	2,704	782	786	85	151	6,754
40	1	1,548	698	2,704	11,502	12,881	86	166	29,584
40	3	1,548	698	2,704	3,879	4,354	86	166	13,434
40	4	1,548	698	2,704	2,926	3,288	86	166	11,415
40	8	1,548	698	2,704	1,496	1,689	86	166	8,387
40	12	1,548	698	2,704	1,020	1,156	86	166	7,377
40	16	1,548	698	2,704	782	889	86	166	6,872
45	1	1,548	698	2,704	11,502	14,515	86	181	31,234
45	2	1,548	698	2,704	5,784	7,303	86	181	18,305
45	4	1,548	698	2,704	2,926	3,697	86	181	11,840
45	8	1,548	698	2,704	1,496	1,894	86	181	8,608
45	12	1,548	698	2,704	1,020	1,293	86	181	7,531
45	16	1,548	698	2,704	782	993	86	181	6,992

All sizes in MBs.

## Supporting Information

Data S1 **Supplementary material.**
(PDF)
