# Correction: Indexes of Large Genome Collections on a PC

**DOI:** 10.1371/journal.pone.0128172

**Published:** 2015-05-08

**Authors:** Agnieszka Danek, Sebastian Deorowicz, Szymon Grabowski

There is an error in the first sentence of the Funding section. The correct sentence is: The work was supported by the National Science Centre under the project DEC-2013/09/B/ST6/03117 and European Social Fund project UDA-POKL.04.01.01-00-106/09.

The correct, complete Funding section should read: The work was supported by the National Science Centre under the project DEC-2013/09/B/ST6/03117 and European Social Fund project UDA-POKL.04.01.01-00-106/09. The work was performed using the infrastructure supported by POIG.02.03.01-24-099/13 grant: “GeCONiI---Upper Silesian Center for Computational Science and Engineering”. The funders had no role in study design, data collection and analysis, decision to publish, or preparation of the manuscript.
