# Supplementary material for: Indexes of Large Genome Collections on a PC
Source: PLoS One. 2014 Oct 7;9(10):e109384. doi: 10.1371/journal.pone.0109384 (PMC4188820; doi:10.1371/journal.pone.0109384)
Supplement: Data S1 — Supplementary material. (PDF) [file pone.0109384.s001.pdf]

Data S1

This supplementary material describes all the necessary steps to repeat the experiments described in the main paper. The package with all source codes can be downloaded from: http://sun.aei.polsl.pl/mugi/. Additional tables and figures are also included.

1 Mulitple Genomes Index description

Multiple Genome Index is a compressed index of collections of genomes of the same species. It allows to ask for both exact and approximate queries.

Here we describe the usage of the tools to build the index (MuGI_build), perform the search (MuGI_search), and parse the result to a readable form (MuGI_parse). We also provide a code generating example queries (MuGI_exGen). All these tools are implemented in C/C++.

1.1 MuGI_build

The program builds the index with set sparsity and length of k-mers, based on the input reference sequence and Variant Call Format (VCF) file describing the differences between individual genomes and reference genome.

Usage:

- MuGI_build <input.fasta> <input.vcf> <k> <sp> <pl>

Parameters:

- input.fasta — name of the input FASTA file with the reference
- input.vcf — name of the input VCF file
- k — desired length of k-mers in the index
- sp — desired sparsity of the index
- pl — ploidity of the individuals in the collection (only 1 and 2 supported)

The output files:

- index-<k>-<sp> — built index
- index-dict — dictionary file

The following are required to build the sources:

- Intel Threading Building Blocks library (https://www.threadingbuildingblocks.org)
- Compiler with OpenMP support (http://openmp.org)

If the ploidity is set to 2, any haploid genotype call is treated as diploid. The diploid genotypes should be phased. The first word in successive chromosome headers should match successive chromosomes described in the VCF file (its first column). The positions of the variants in the VCF file should be within the current chromosome (first column). During the process they are adjusted to present the position within the whole reference based on the size of chromosomes sequences. The index-dict file is created along with the index. It contains data about sizes of all chromosomes and names of all individuals. It is used to parse the search results to a readable form.

For testing we implemented the possibility to build few indexes with the same k-mer length and different sparsities (from 1 up to $< sp >$) at the same run. To use this mode, `CREATE_ALL_INDEXES` must be defined in the `defs.h` file (see line 14 of `defs.h`).

1.2 MuGI_search

The program performs the search of the input queries in the compressed collection (input index file) and reports to the stdout statistics about queries execution times. Asmlib library (http://www.agner.org/optimize/asmlib.zip) is required to build the sources.

Usage:

- `MuGI_search <index-file> <queries-file> {<maxError>}`

Parameters:

- `index-file` — path to file with the index
- `queries-file` — path to file with the sequences to search (FASTA or FASTQ or plain sequences - one in each line)
- `maxError` — optional, maximum number of mismatches in found sequence (default 0, exact search)

The output file:

- `result.out` — binary file with the result

1.3 MuGI_parse

The program parses the binary file with result (output of `MuGI_search`) and creates a text file with readable data.

For each queries, at first its name (for FASTQ and FASTA) or ordinal number (for simple list) is written. Then, all found positions are reported (chromosome number and position within it) along with list of all individuals in which the match was found. In a ”hex” mode (0), the list is represented by a bit vector written in a hexadecimal form, where each jth bit set corresponds to match found in jth haploid genome. In the ”full” mode (1) the list consist of names of all individuals with the match. For diploid individuals, to distinguish between two haploid sequences of each, a suffix ”-1” or ”-2” is added to the name. In the bit vector representation, each diploid individual is represented by two consecutive bits.

Usage:

- `MuGI_parse <dict> <file_to_parse> <output_file> <mode>`

Parameters:

- `dict` — dictionary file of the index
- `file_to_parse` — binary file with the result
- `output_file` — name of the output file
- `mode` — optional, mode of the output: 0 (hexadecimal individuals representation) or 1 (full individual names) default: 0

1.4 MuGI_genEx

The program generates a file with a set number of queries. The file an be in FASTQ or FASTA format, or be a text file with list of sequences (three acceptable formats of queries for `MuGI_search`). It uses the created index file, to take an excerpts from the collection of genomes. As only Reference (REF), Variant Database (VD) and Bit Vectors (BV) are used, the k and sp parameters of the index building process does not matter. The final queries are made as described in the main paper.

Usage:

- `MuGI_genEx <index-file> <out-file> <no_reads> {<mode>}`

Parameters:

- `index-file` — path to file with the index
- `out-file` — output file with example reads
- `no_reads` — number of reads to generate
- `mode` — optional, output format: "fastq" or "fasta" or "list" (default: "list")

2 Data source

The data used in experiments are from the Phase 1 of the 1000 Genomes Project, which contains data about 1092 human individuals.

The human reference sequence used can be found at the NCBI's anonymous FTP server (ftp://ftp-trace.ncbi.nih.gov//1000genomes/ftp/technical/reference//human_g1k_v37.fasta.gz). We removed all non-chromosomal supercontigs, leaving only 1–22, X, and Y chromosomes.

The VCF (Variant Call Format) files can be downloaded from the NCBI's anonymous FTP server. It can be either EBI (ftp://ftp.1000genomes.ebi.ac.uk/vol1/ftp/phase1/analysis_results/integrated_call_sets/) or NCBI (ftp://ftp.ncbi.nih.gov//1000genomes/ftp/phase1/analysis_results/integrated_call_sets/) FTP site. Note that the size of the complete set is about 1.2 TB. The complete list of compressed VCF files used:

```
ALL.chr1.integrated_phase1_v3.20101123.snps_indels_svs.genotypes.vcf.gz
ALL.chr2.integrated_phase1_v3.20101123.snps_indels_svs.genotypes.vcf.gz
ALL.chr3.integrated_phase1_v3.20101123.snps_indels_svs.genotypes.vcf.gz
ALL.chr4.integrated_phase1_v3.20101123.snps_indels_svs.genotypes.vcf.gz
ALL.chr5.integrated_phase1_v3.20101123.snps_indels_svs.genotypes.vcf.gz
ALL.chr6.integrated_phase1_v3.20101123.snps_indels_svs.genotypes.vcf.gz
ALL.chr7.integrated_phase1_v3.20101123.snps_indels_svs.genotypes.vcf.gz
ALL.chr8.integrated_phase1_v3.20101123.snps_indels_svs.genotypes.vcf.gz
ALL.chr9.integrated_phase1_v3.20101123.snps_indels_svs.genotypes.vcf.gz
ALL.chr10.integrated_phase1_v3.20101123.snps_indels_svs.genotypes.vcf.gz
ALL.chr11.integrated_phase1_v3.20101123.snps_indels_svs.genotypes.vcf.gz
ALL.chr12.integrated_phase1_v3.20101123.snps_indels_svs.genotypes.vcf.gz
ALL.chr13.integrated_phase1_v3.20101123.snps_indels_svs.genotypes.vcf.gz
ALL.chr14.integrated_phase1_v3.20101123.snps_indels_svs.genotypes.vcf.gz
ALL.chr15.integrated_phase1_v3.20101123.snps_indels_svs.genotypes.vcf.gz
ALL.chr16.integrated_phase1_v3.20101123.snps_indels_svs.genotypes.vcf.gz
ALL.chr17.integrated_phase1_v3.20101123.snps_indels_svs.genotypes.vcf.gz
ALL.chr18.integrated_phase1_v3.20101123.snps_indels_svs.genotypes.vcf.gz
```

```
ALL.chr19.integrated_phase1_v3.20101123.snps_indels_svs.genotypes.vcf.gz
ALL.chr20.integrated_phase1_v3.20101123.snps_indels_svs.genotypes.vcf.gz
ALL.chr21.integrated_phase1_v3.20101123.snps_indels_svs.genotypes.vcf.gz
ALL.chr22.integrated_phase1_v3.20101123.snps_indels_svs.genotypes.vcf.gz
ALL.chrX.integrated_phase1_v3.20101123.snps_indels_svs.genotypes.vcf.gz
ALL.chrY.phase1_samtools_si.20101123.snps.low_coverage.genotypes.vcf.gz
```

Because of the two pseudoautosomal regions (PAR1 and PAR2) shared between X and Y chromosomes in male individuals, the diploid genotype calls for variants in the pseudoautosomal regions in X and Y chromosomes are stored in the available VCF file for X chromosome, while haploid genotype calls for non-pseudoautosomal regions (nonPAR, between PAR1 and PAR2) of X and Y chromosomes are stored in the corresponding VCF files. For Y chromosome there are information for one additional individual (not present in other VCF files). To have consistent data with one diploid representation for the whole reference, we removed the extra individual data and artificially extended the list of genomes described in the VCF file of chromosome Y to be the same as for other chromosomes, that is, to include female individuals. The alleles values were set to zero (reference allele) in the genotype fields. The tool building the index with the diploidity parameter is set to 2, treats all haploid calls as diploid—occurring variant is introduced in two generated sequences. This modification of the dataset means there can be matches reported in non existing regions, i.e, chromosome Y of a female individual or two chromosomes X of a male individual, but these false results are easy to identify and correct in the post processing process.

To build an index for a single chromosome of all individuals in the collection, the reference sequences of single assembled chromosomes may be useful. They can be found at the GenBank and downloaded from the NCBI's anonymous FTP server:
ftp://ftp.ncbi.nlm.nih.gov/genbank/genomes/Eukaryotes/vertebrates_mammals/Homo_sapiens/GRCh37/Primary_Assembly/assembled_chromosomes/FASTA/

3 Experiments

Here we describe all necessary steps to build an index and perform experiments on single human chromosome and on the whole human genome.

3.1 Single chromosome

To build the index for a single autosomal chromosome of all individuals from the collection, it is enough to download the related reference sequence and VCF file. Only FASTA file requires slight modification, so its first word matches `CHROM` field in the VCF file. The script below shows the steps for the 22 chromosome (`wget` and `gzip` tools required). It downloads and decompresses the data, builds the index with sparsity 3 and k-mer length set to 25, generates 100K queries (in FASTA format), performs exact search and parse the resulting file to store the complete report (positions and names of individuals) in the `full-results.txt` file

```
wget ftp://ftp.1000genomes.ebi.ac.uk/vol1/ftp/phase1/analysis_results/
    integrated_call_sets/ALL.chr22.integrated_phase1_v3.20101123.
    snps_indels_svs.genotypes.vcf.gz
gzip -d ALL.chr22.integrated_phase1_v3.20101123.snps_indels_svs.genotypes.vcf.gz
mv ALL.chr22.integrated_phase1_v3.20101123.snps_indels_svs.genotypes.vcf > chr22.vcf
wget ftp://ftp.ncbi.nlm.nih.gov/genbank/genomes/Eukaryotes/vertebrates_mammals/
    Homo_sapiens/GRCh37/Primary_Assembly/assembled_chromosomes/FASTA/chr22.fa.gz
gzip -d chr22.fa.gz
```

```
sed 's/>/>22 /g' chr22.fa > chr22.fasta
./MuGI_build chr22.fasta chr22.vcf 25 3 2
./MuGI_genEx index-25-3 queries.fa 100000 fasta
./MuGI_search index-25-3 queries.fa 0
./MuGI_parse results.out full-results.txt 1
```

3.2 Whole genome

Building the index for the whole genome requires pre-processing of the available data (reference sequence and the 24 VCF files) as described in Section 2. The detailed steps and additional guidelines (for the unix-like environment):

1. Pre-processing the reference sequence

   We cut from the downloaded and decompressed reference file anything after the Y chromosome (strting from the mitochondria). The following example script downloads the available reference and creates `human.fasta` file with the desired reference (`wget`, `gzip` and `awk` tools required):

   ```
   wget ftp://ftp-trace.ncbi.nih.gov/1000genomes/ftp/technical/reference/
       human_g1k_v37.fasta.gz
   gzip -d human_g1k_v37.fasta.gz
   awk 'BEGIN{c=1} /^>MT/ {c=0} // {if(c==1){print $0}}' human_g1k_v37.fasta > human.fasta
   ```

2. Pre-processing the Y chromosome

   For consistency of the data the VCF file for the Y chromosome were pre-processed as described in Section 2. We are providing the C++ example code `preProcessY.cpp` together with MuGI distribution, that can be used for this step. The arguments of the program are: input Y chromosome VCF file and output name. The following example script downloads and pre-processes the VCF file and creates its modified version: `chrY.vcf` (gcc compiler used in the example, `wget` and `gzip` tools required):

   ```
   wget ftp://ftp.1000genomes.ebi.ac.uk/vol1/ftp/phase1/analysis_results/
       integrated_call_sets/ALL.chrY.phase1_samtools_si.20101123.snps.
       low_coverage.genotypes.vcf.gz
   gzip -d ALL.chrY.phase1_samtools_si.20101123.snps.low_coverage.genotypes.vcf.gz
   g++ -O3 preProcessY/preProcessY.cpp -o prepY
   ./prepY ALL.chrY.phase1_samtools_si.20101123.snps.low_coverage.genotypes.vcf chrY.vcf
   ```

3. Merging the VCF files

   We merged all 24 VCF files downloaded from the data source (section 2), including pre-processed VCF file for Y chromosome (previous step) to one VCF file with single header (taken from the VCF file of 1 chromosome, other headers were discarded). The example script performing the task (assuming all VCF files are already decompressed and the modified VCF file name is `chrY.vcf`) and creating single `human.vcf` file:

   ```
   cp ALL.chr1.integrated_phase1_v3.20101123.snps_indels_svs.genotypes.vcf human.vcf
   tail -n+31 ALL.chr2.integrated_phase1_v3.20101123.snps_indels_svs.genotypes.vcf >> human.vcf
   tail -n+31 ALL.chr3.integrated_phase1_v3.20101123.snps_indels_svs.genotypes.vcf >> human.vcf
   tail -n+31 ALL.chr4.integrated_phase1_v3.20101123.snps_indels_svs.genotypes.vcf >> human.vcf
   tail -n+31 ALL.chr5.integrated_phase1_v3.20101123.snps_indels_svs.genotypes.vcf >> human.vcf
   tail -n+31 ALL.chr6.integrated_phase1_v3.20101123.snps_indels_svs.genotypes.vcf >> human.vcf
   tail -n+31 ALL.chr7.integrated_phase1_v3.20101123.snps_indels_svs.genotypes.vcf >> human.vcf
   ```

```
tail -n+31 ALL.chr8.integrated_phase1_v3.20101123.snps_indels_svs.genotypes.vcf >> human.vcf
tail -n+31 ALL.chr9.integrated_phase1_v3.20101123.snps_indels_svs.genotypes.vcf >> human.vcf
tail -n+31 ALL.chr10.integrated_phase1_v3.20101123.snps_indels_svs.genotypes.vcf >> human.vcf
tail -n+31 ALL.chr11.integrated_phase1_v3.20101123.snps_indels_svs.genotypes.vcf >> human.vcf
tail -n+31 ALL.chr12.integrated_phase1_v3.20101123.snps_indels_svs.genotypes.vcf >> human.vcf
tail -n+31 ALL.chr13.integrated_phase1_v3.20101123.snps_indels_svs.genotypes.vcf >> human.vcf
tail -n+31 ALL.chr14.integrated_phase1_v3.20101123.snps_indels_svs.genotypes.vcf >> human.vcf
tail -n+31 ALL.chr15.integrated_phase1_v3.20101123.snps_indels_svs.genotypes.vcf >> human.vcf
tail -n+31 ALL.chr16.integrated_phase1_v3.20101123.snps_indels_svs.genotypes.vcf >> human.vcf
tail -n+31 ALL.chr17.integrated_phase1_v3.20101123.snps_indels_svs.genotypes.vcf >> human.vcf
tail -n+31 ALL.chr18.integrated_phase1_v3.20101123.snps_indels_svs.genotypes.vcf >> human.vcf
tail -n+31 ALL.chr19.integrated_phase1_v3.20101123.snps_indels_svs.genotypes.vcf >> human.vcf
tail -n+31 ALL.chr20.integrated_phase1_v3.20101123.snps_indels_svs.genotypes.vcf >> human.vcf
tail -n+31 ALL.chr21.integrated_phase1_v3.20101123.snps_indels_svs.genotypes.vcf >> human.vcf
tail -n+31 ALL.chr22.integrated_phase1_v3.20101123.snps_indels_svs.genotypes.vcf >> human.vcf
tail -n+32 ALL.chrX.integrated_phase1_v3.20101123.snps_indels_svs.genotypes.vcf >> human.vcf
tail -n+27 ALL.chrY.vcf >> human.vcf
```

In our experiments we merged the minimal versions of the VCF files (VCFmin files, that can be obtained from the VCF files as in [Deorowicz *et al.*(2013)]), what speeds up the merging and reduces the required disk space. The resulting `human.vcf` file is a valid input for the `MuGI_build` program.

4. Building the index

   To build the index with k-mer length 30 and sparsity 4:

   ```
   ./MuGI_bulid human.fasta human.vcf 30 4 2
   ```

   The resulting index name is `index-30-4`

5. Generating the sample simulated data

   To generate 100K sample queries for the collection, stored as a simple list:

   ```
   ./MuGI_genEx index-30-4 queries.txt 100000
   ```

   The reads (simulated and real) used in the experiments can be downloaded from sun.aei.polsl.pl/mugi.

6. Searching

   Example search for generated sequences in the compressed collection, assuming maximum 2 mismatches:

   ```
   ./MuGI_search index-30-4 queries.txt 2
   ```

   The result is stored in the binary file `result.out`

7. Parsing the result

   To see the results in a readble format (with hexadecimal representation of occurrence or not of match in all individuals):

   ```
   ./MuGI_parse index-dict result.out parsed_results.txt 0
   ```

References

[Deorowicz *et al.*(2013)] Deorowicz, S. *et al.* (2013) Genome compression: a novel approach for large collections. *Bioinformatics*, **29**(20), 2572–2578.

4 Additional figures

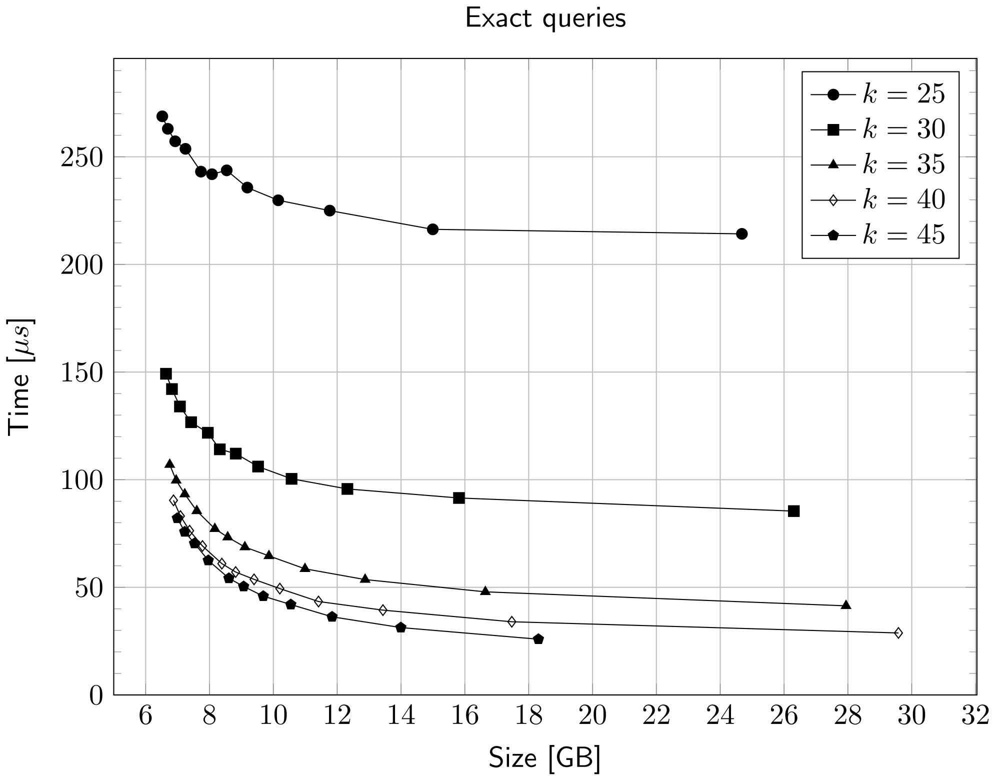

Figure 1: Times for exact queries for various k and *sparsity*. The rightmost points for each k is for *sparsity* = 1 except for k = 45, where the rightmost point is for *sparsity* = 2 (due to limitation of our test machine)

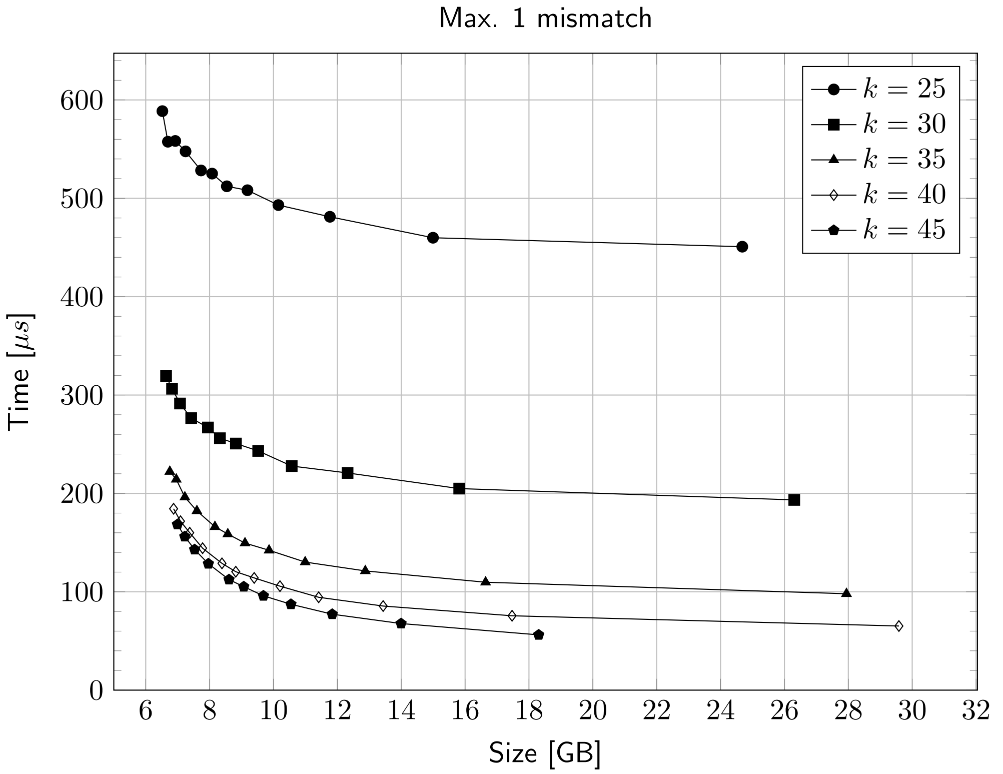

Figure 2: Times for up to 1 mismatch queries for various k and *sparsity*. The rightmost points for each k is for $sparsity = 1$ except for $k = 45$, where the rightmost point is for $sparsity = 2$ (due to limitation of our test machine)

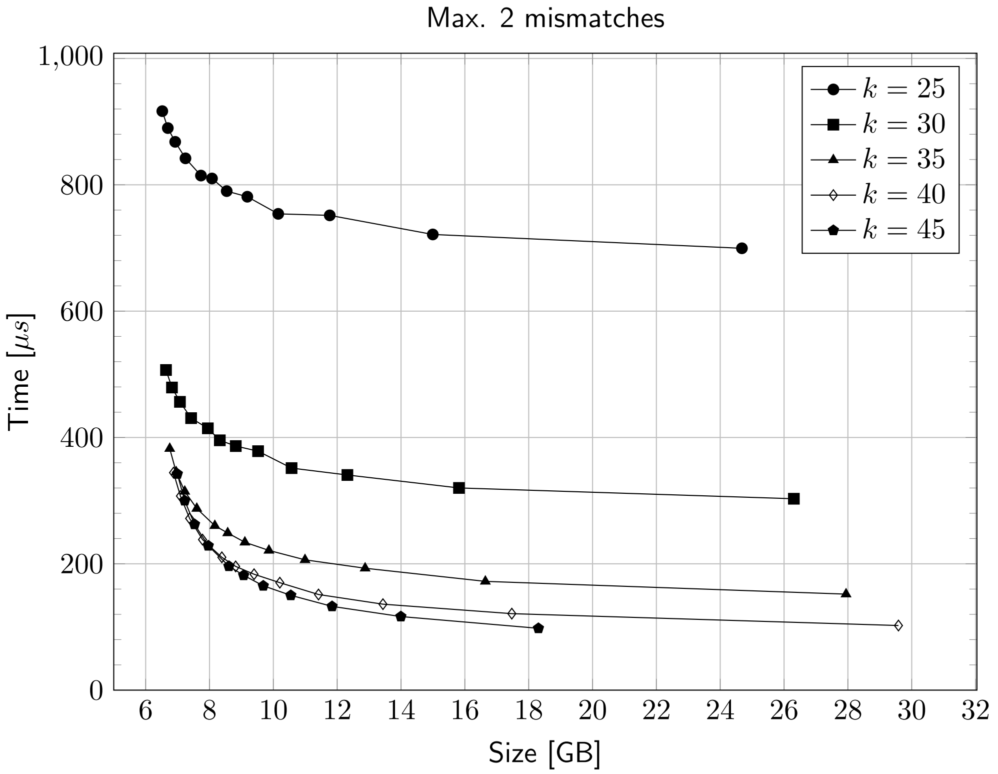

Figure 3: Times for up to 2 mismatches queries for various k and *sparsity*. The rightmost points for each k is for $sparsity = 1$ except for $k = 45$, where the rightmost point is for $sparsity = 2$ (due to limitation of our test machine)

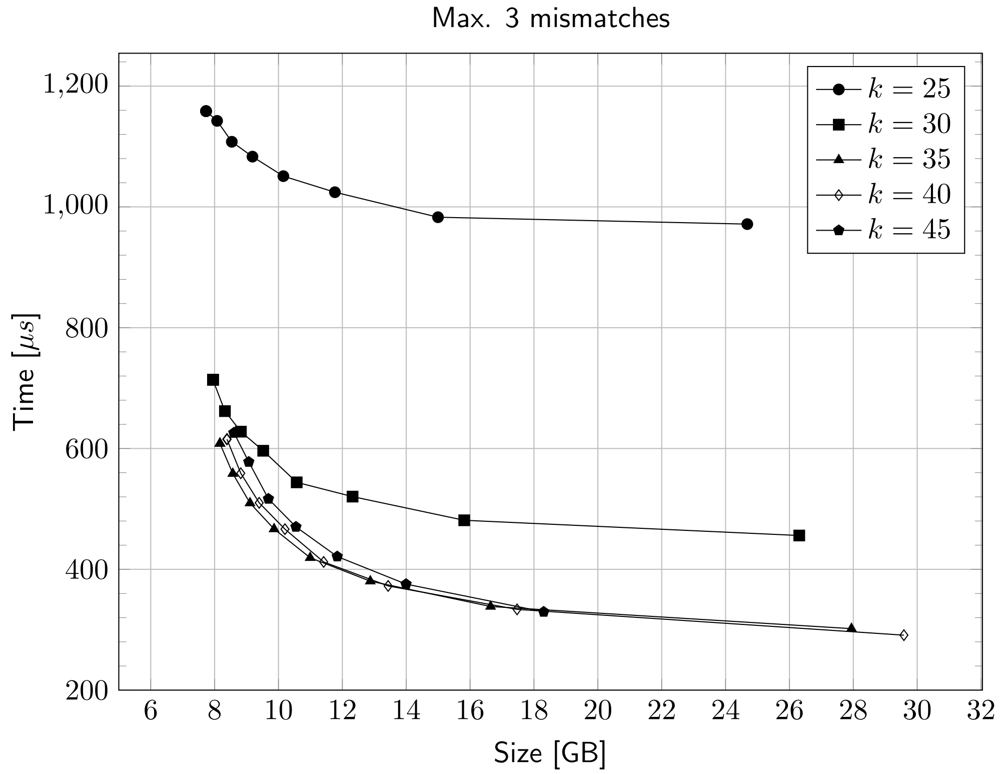

Figure 4: Times for up to 3 mismatches queries for various k and *sparsity*. The rightmost points for each k is for *sparsity* $= 1$ except for $k = 45$, where the rightmost point is for *sparsity* $= 2$ (due to limitation of our test machine)

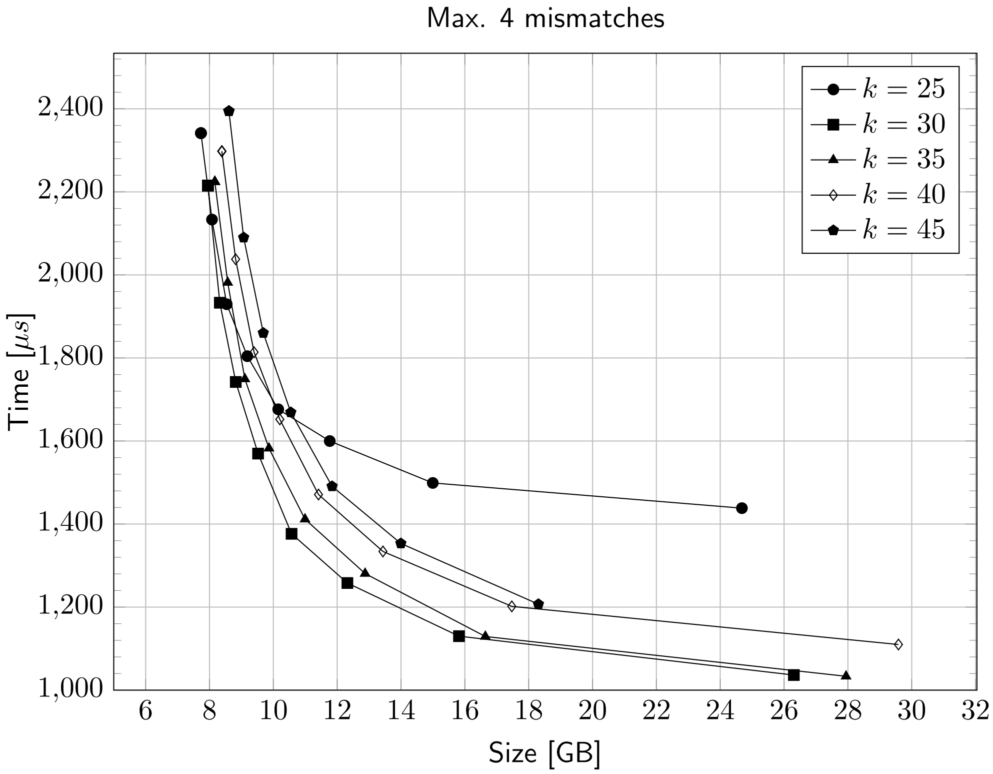

Figure 5: Times for up to 4 mismatches queries for various k and *sparsity*. The rightmost points for each k is for $sparsity = 1$ except for $k = 45$, where the rightmost point is for $sparsity = 2$ (due to limitation of our test machine)

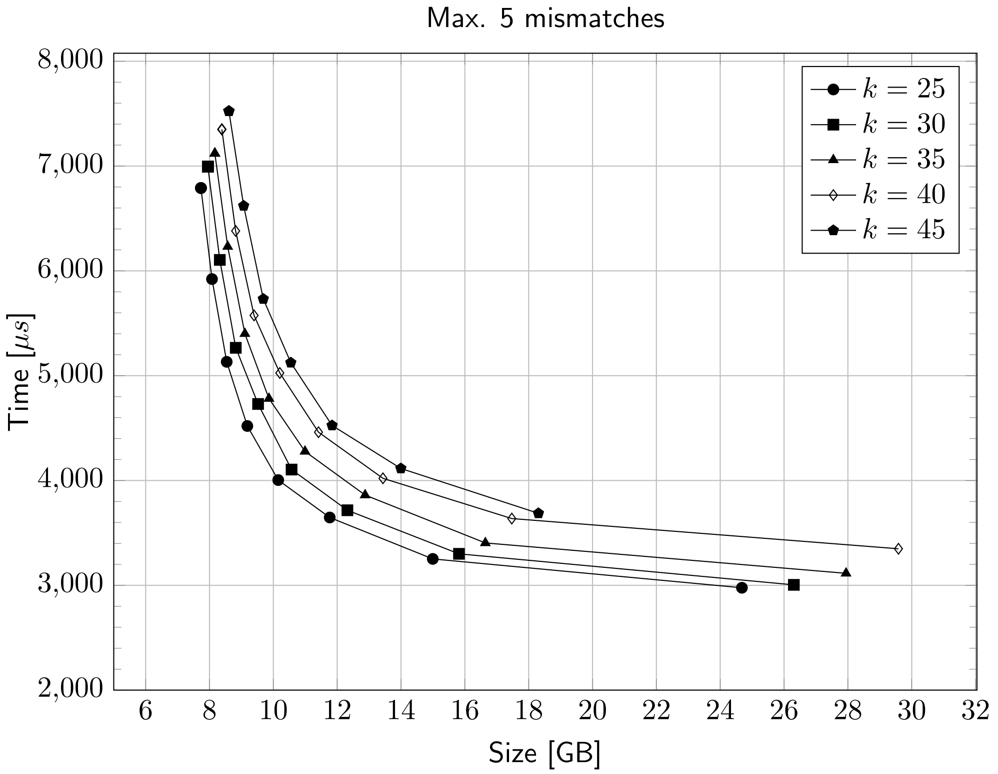

Figure 6: Times for up to 5 mismatches queries for various k and *sparsity*. The rightmost points for each k is for *sparsity* $= 1$ except for $k = 45$, where the rightmost point is for *sparsity* $= 2$ (due to limitation of our test machine)

$k = 40$, $sparsity = 3$

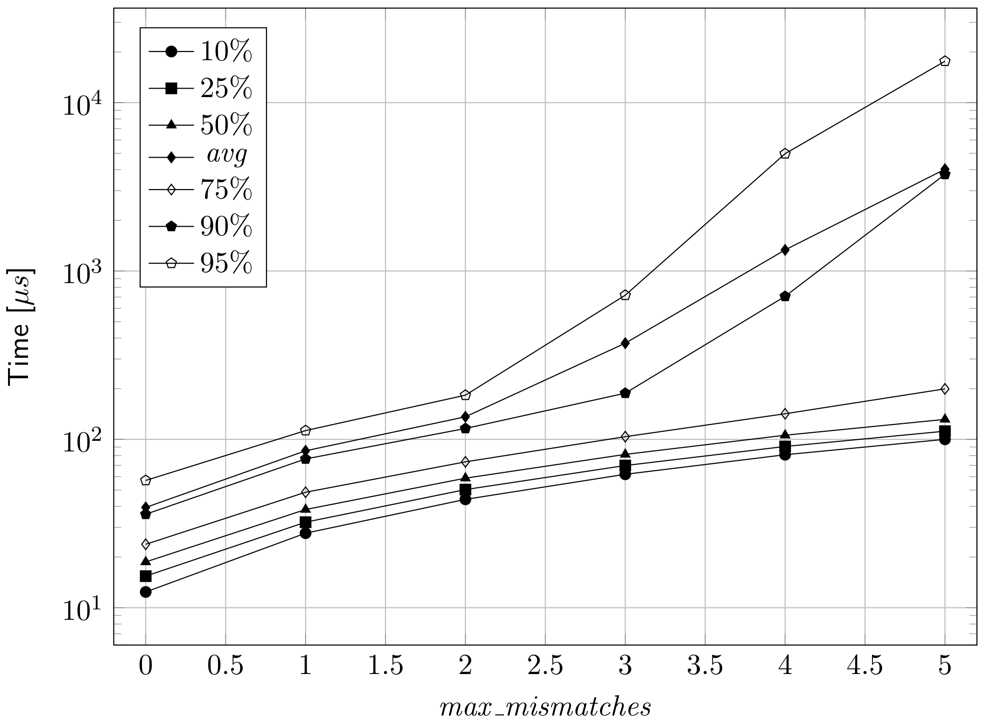

Figure 7: Percentile times for various types of queries (from 0 to 5 mismatches)

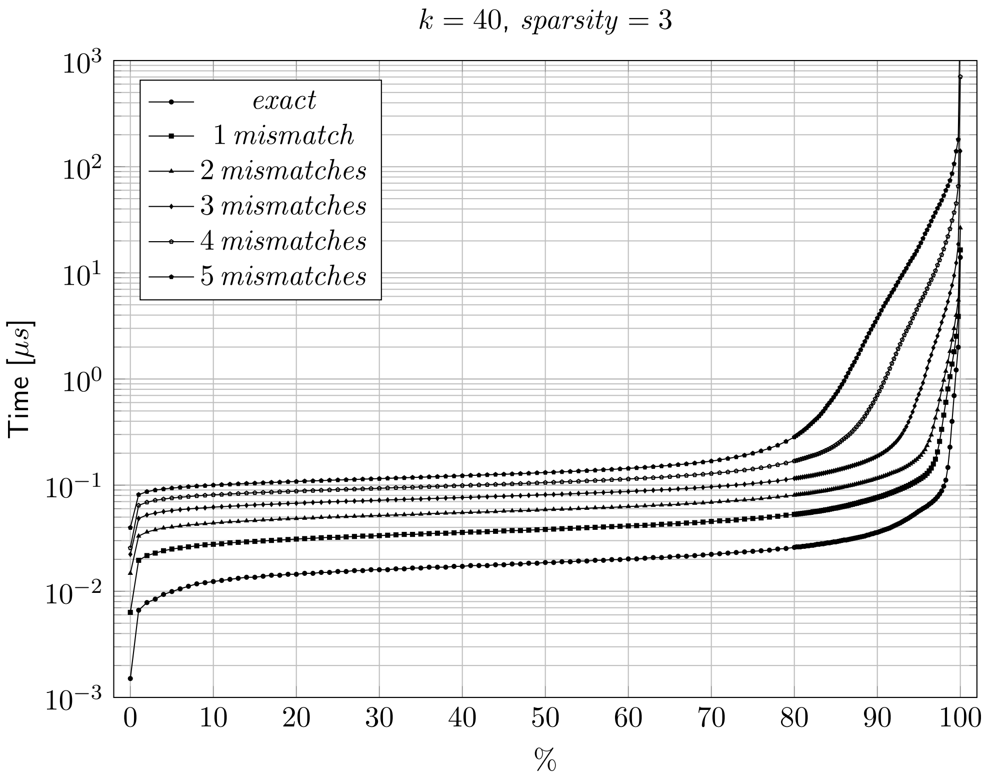

Figure 8: Percentile times for various types of queries (from 0 to 5 mismatches)

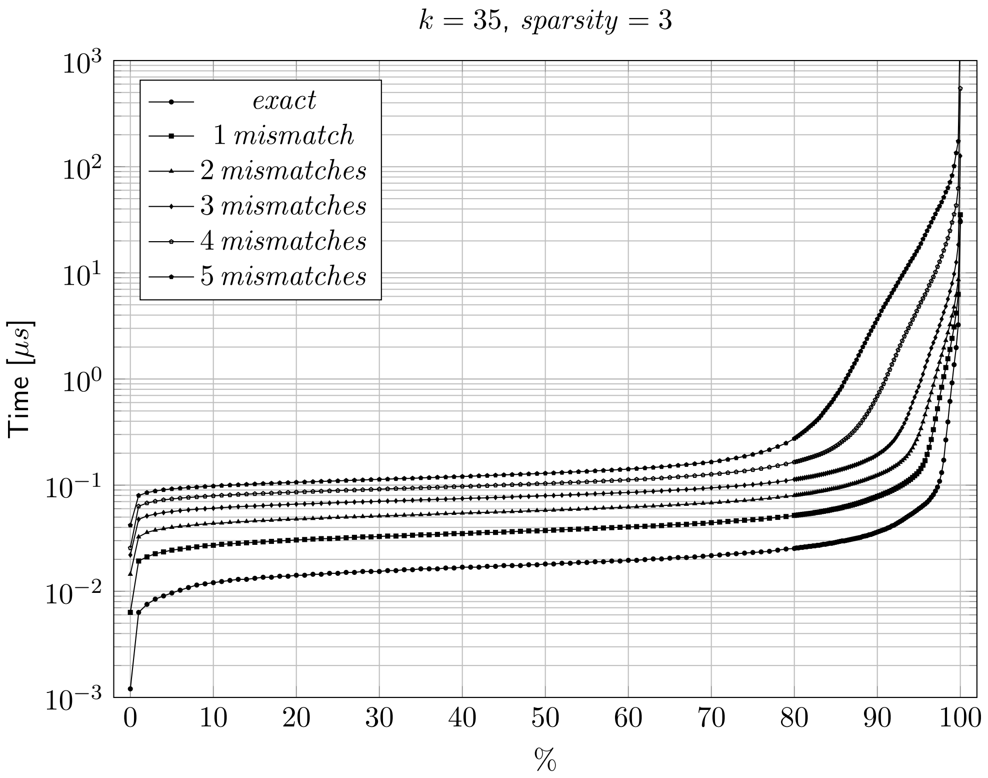

Figure 9: Percentile times for various types of queries (from 0 to 5 mismatches)

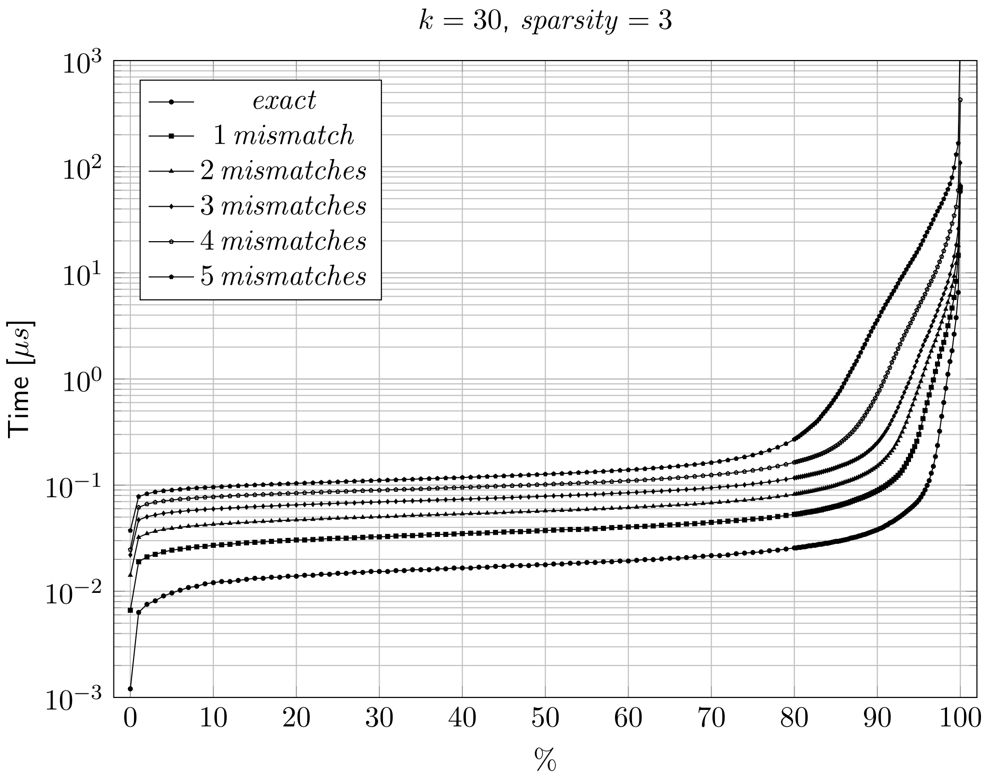

Figure 10: Percentile times for various types of queries (from 0 to 5 mismatches)

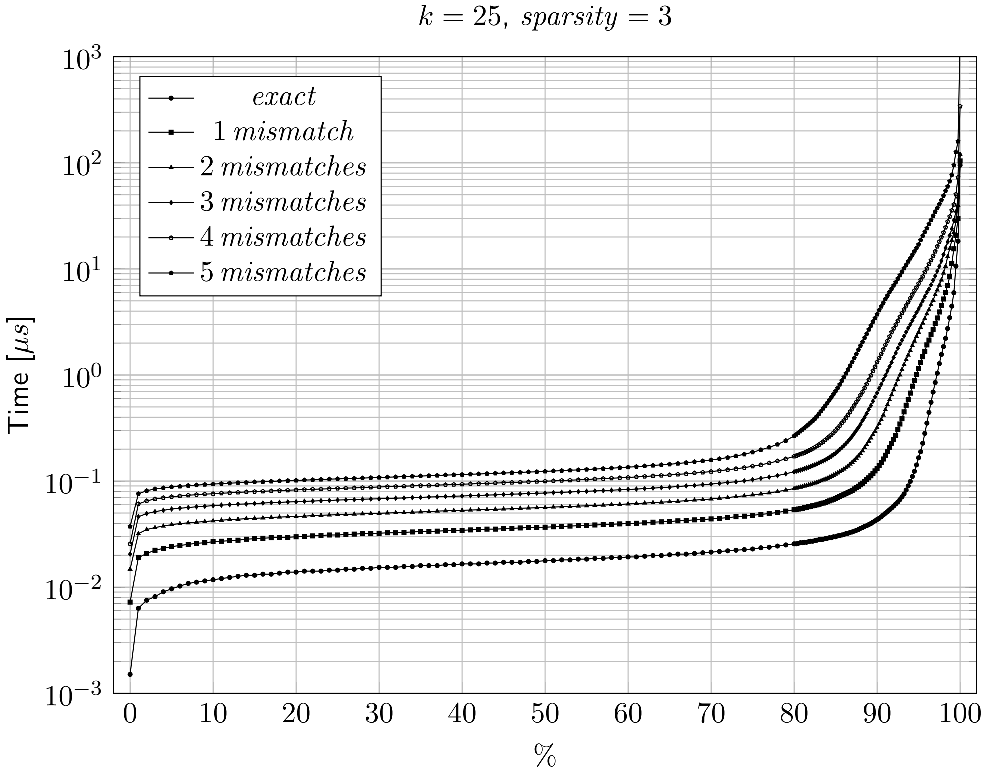

Figure 11: Percentile times for various types of queries (from 0 to 5 mismatches)

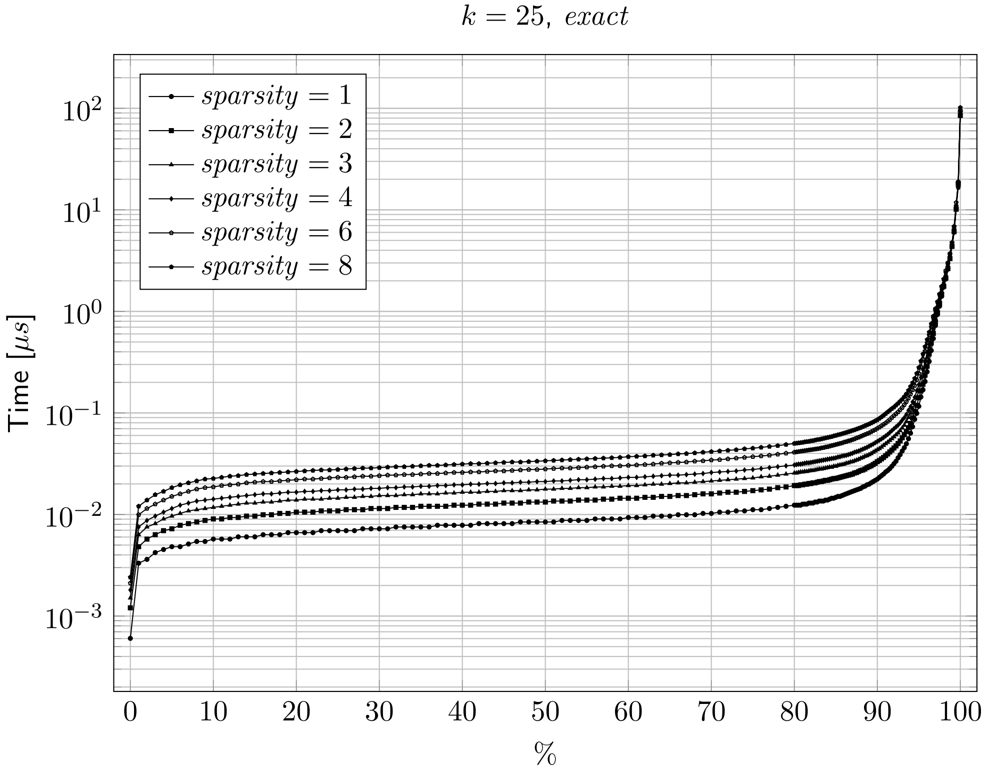

Figure 12: Percentile times for various types of queries (from 0 to 5 mismatches)

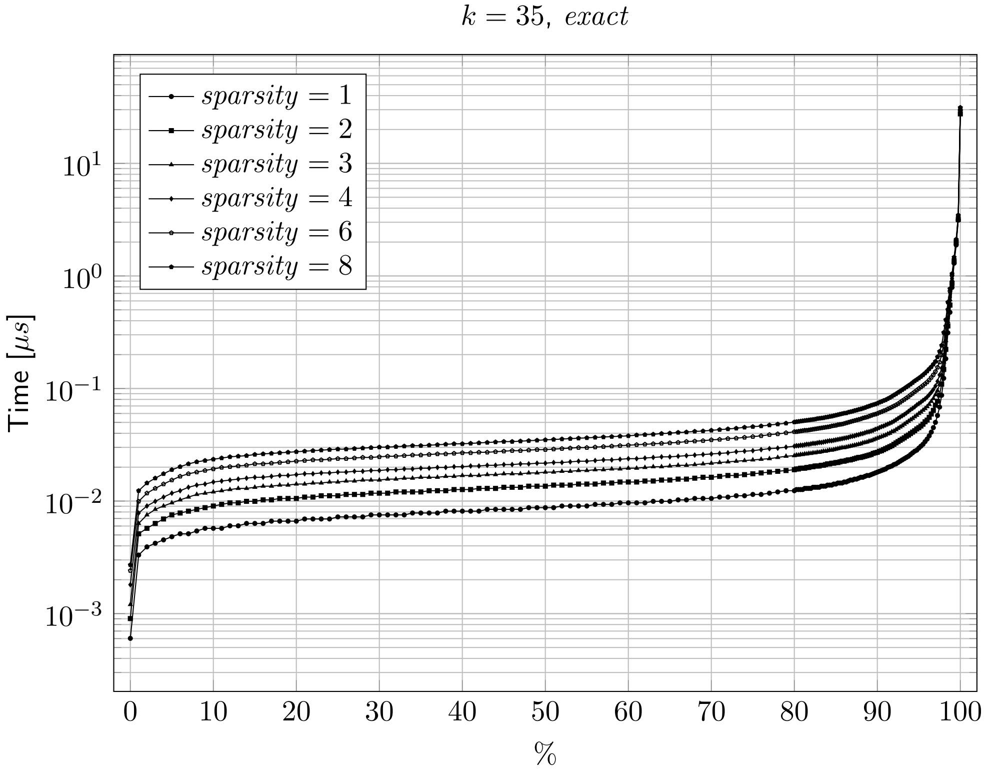

Figure 13: Percentile times for various types of queries (from 0 to 5 mismatches)

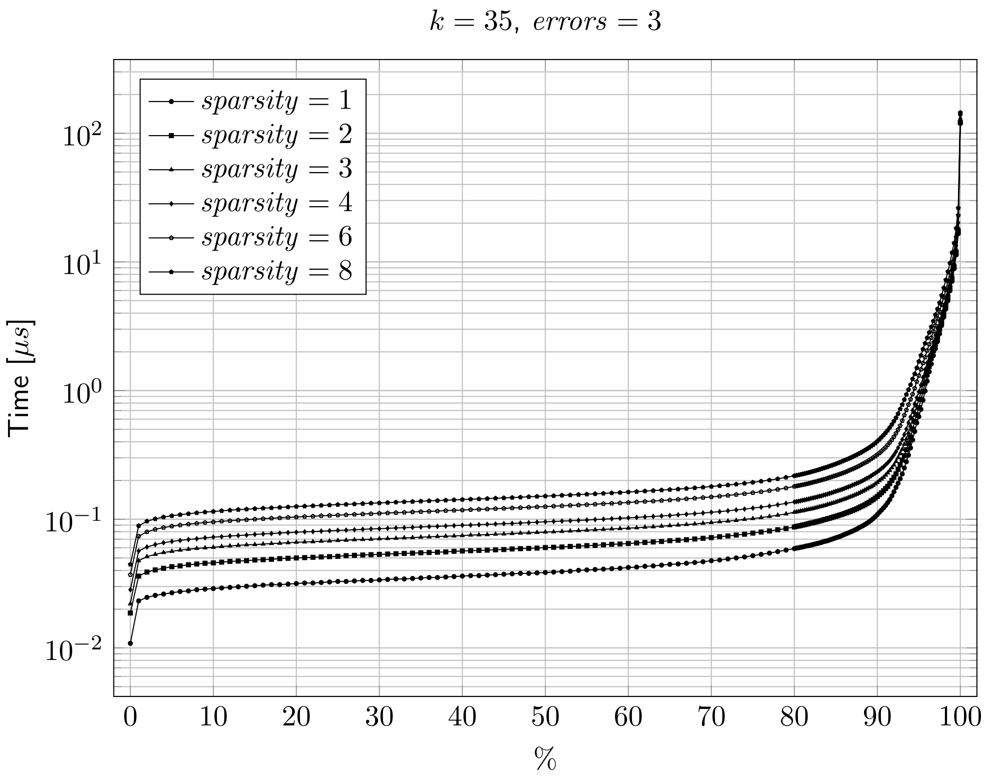

Figure 14: Percentile times for various types of queries (from 0 to 5 mismatches)

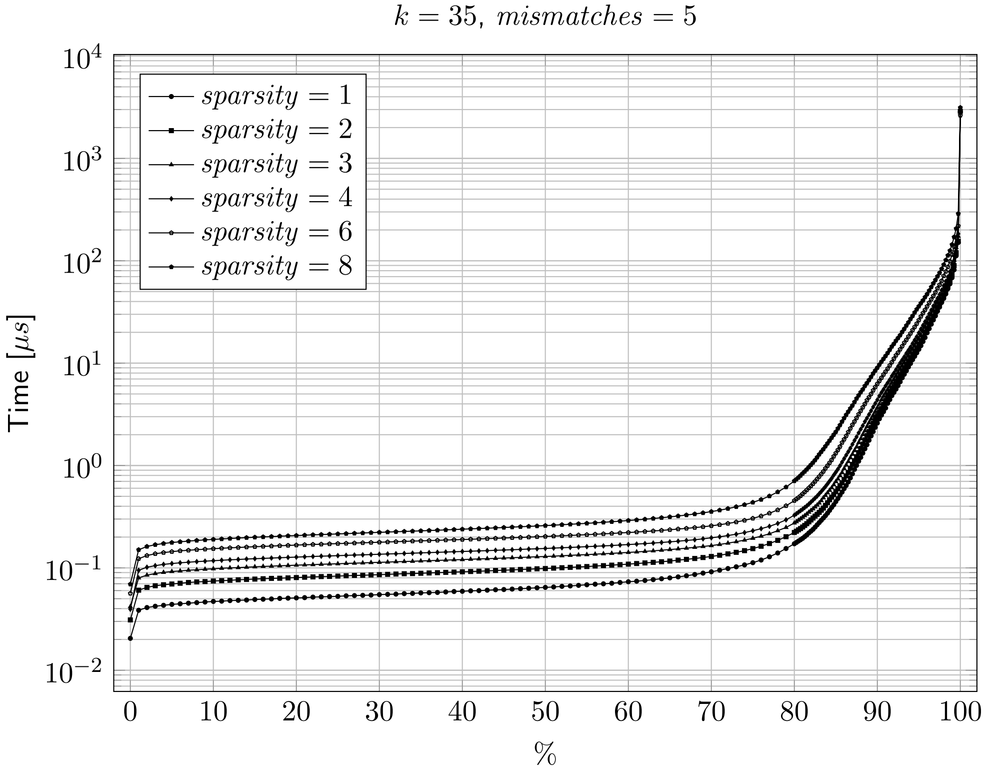

Figure 15: Percentile times for various types of queries (from 0 to 5 mismatches)
